# Nonlinear dynamics of multi-omics profiles during human aging

**DOI:** 10.1038/s43587-024-00692-2

**Published:** 2024-08-14

**Authors:** Xiaotao Shen, Chuchu Wang, Xin Zhou, Wenyu Zhou, Daniel Hornburg, Si Wu, Michael P. Snyder

**Affiliations:** 1 Department of Genetics, Stanford University School of Medicine, Stanford, CA USA; 2 Lee Kong Chian School of Medicine, Nanyang Technological University, Singapore, Singapore; 3 School of Chemistry, Chemical Engineering and Biotechnology, Singapore, Singapore; 4 Howard Hughes Medical Institute, Stanford University, Stanford, CA USA; 5 Department of Molecular and Cellular Physiology, Stanford University, Stanford, CA USA; 6 Stanford Center for Genomics and Personalized Medicine, Stanford, CA USA

**Keywords:** Systems biology, Biochemistry, Ageing

## Abstract

Aging is a complex process associated with nearly all diseases. Understanding the molecular changes underlying aging and identifying therapeutic targets for aging-related diseases are crucial for increasing healthspan. Although many studies have explored linear changes during aging, the prevalence of aging-related diseases and mortality risk accelerates after specific time points, indicating the importance of studying nonlinear molecular changes. In this study, we performed comprehensive multi-omics profiling on a longitudinal human cohort of 108 participants, aged between 25 years and 75 years. The participants resided in California, United States, and were tracked for a median period of 1.7 years, with a maximum follow-up duration of 6.8 years. The analysis revealed consistent nonlinear patterns in molecular markers of aging, with substantial dysregulation occurring at two major periods occurring at approximately 44 years and 60 years of chronological age. Distinct molecules and functional pathways associated with these periods were also identified, such as immune regulation and carbohydrate metabolism that shifted during the 60-year transition and cardiovascular disease, lipid and alcohol metabolism changes at the 40-year transition. Overall, this research demonstrates that functions and risks of aging-related diseases change nonlinearly across the human lifespan and provides insights into the molecular and biological pathways involved in these changes.

## Abstract (web summary)

Understanding the molecular changes underlying aging is important for developing biomarkers and healthy aging interventions. In this study, the authors used comprehensive multi-omics data to reveal nonlinear molecular profiles across chronological ages, highlighting two substantial variations observed around ages 40 and 60, which are linked to increased disease risks.

## Main

Aging is a complex and multifactorial process of physiological changes strongly associated with various human diseases, including cardiovascular diseases (CVDs), diabetes, neurodegeneration and cancer^1^. The alterations of molecules (including transcripts, proteins, metabolites and cytokines) are critically important to understand the underlying mechanism of aging and discover potential therapeutic targets for aging-related diseases. Recently, the development of high-throughput omics technologies has enabled researchers to study molecular changes at the system level^2^. A growing number of studies have comprehensively explored the molecular changes that occur during aging using omics profiling^3,4^, and most focus on linear changes^5^. It is widely recognized that the occurrence of aging-related diseases does not follow a proportional increase with age. Instead, the risk of these diseases accelerates at specific points throughout the human lifespan^6^. For example, in the United States, the prevalence of CVDs (encompassing atherosclerosis, stroke and myocardial infarction) is approximately 40% between the ages of 40 and 59, increases to about 75% between 60 and 79 and reaches approximately 86% in individuals older than 80 years^7^. Similarly, also in the United States, the prevalence of neurodegenerative diseases, such as Parkinson’s disease and Alzheimer’s disease, exhibits an upward trend as well as human aging progresses, with distinct turning points occurring around the ages of 40 and 65, respectively^8–10^. Some studies also found that brain aging followed an accelerated decline in flies^11^ and chimpanzees^12^ that lived past middle age and advanced age.

The observation of a nonlinear increase in the prevalence of aging-related diseases implies that the process of human aging is not a simple linear trend. Consequently, investigating the nonlinear changes in molecules will likely reveal previously unreported molecular signatures and mechanistic insights. Some studies examined the nonlinear alterations of molecules during human aging^13^. For instance, nonlinear changes in RNA and protein expression related to aging have been documented^14–16^. Moreover, certain DNA methylation sites have exhibited nonlinear changes in methylation intensity during aging, following a power law pattern^17^. Li et al.^18^ identified the 30s and 50s as transitional periods during women’s aging. Although aging patterns are thought to reflect the underlying biological mechanisms, the comprehensive landscape of nonlinear changes of different types of molecules during aging remains largely unexplored. Remarkably, the global monitoring of nonlinear changing molecular profiles throughout human aging has yet to be fully used to extract basic insights into the biology of aging.

In the present study, we conducted a comprehensive deep multi-omics profiling on a longitudinal human cohort comprising 108 individuals aged from 25 years to 75 years. The cohort was followed over a span of several years (median, 1.7 years), with the longest monitoring period for a single participant reaching 6.8 years (2,471 days). Various types of omics data were collected from the participants’ biological samples, including transcriptomics, proteomics, metabolomics, cytokines, clinical laboratory tests, lipidomics, stool microbiome, skin microbiome, oral microbiome and nasal microbiome. The investigation explored the changes occurring across different omics profiles during human aging. Remarkably, many molecular markers and biological pathways exhibited a nonlinear pattern throughout the aging process, thereby providing valuable insight into periods of dramatic alterations during human aging.

## Results

### Most of the molecules change nonlinearly during aging

**Fig. 1:**
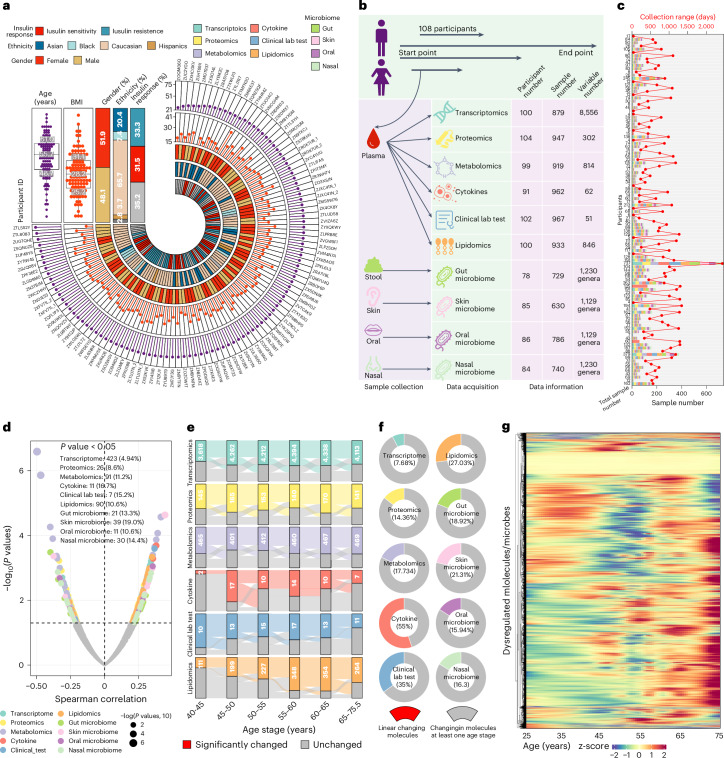
Most molecules and microbes undergo nonlinear changes during human aging. **a**, The demographics of the 108 participants in the study are presented. **b**, Sample collection and multi-omics data acquisition of the cohort. Four types of biological samples were collected, and 10 types of omics data were acquired. **c**, Collection time range and sample numbers for each participant. The top *x* axis represents the collection range for each participant (read line), and the bottom *x* axis represents the sample number for each participant (bar plot). Bars are color-coded by omics type. **d**, Significantly changed molecules and microbes during aging were detected using the Spearman correlation approach (*P* < 0.05). The *P* values were not adjusted (Methods). Dots are color-coded by omics type. **e**, Differential expressional molecules/microbes in different age ranges compared to baseline (25–40 years old, two-sided Wilcoxon test, *P* < 0.05). The *P* values were not adjusted (Methods). **f**, The linear changing molecules comprised only a small part of dysregulated molecules in at least one age range. **g**, Heatmap depicting the nonlinear changing molecules and microbes during human aging.

**Extended Data Fig. 1:**
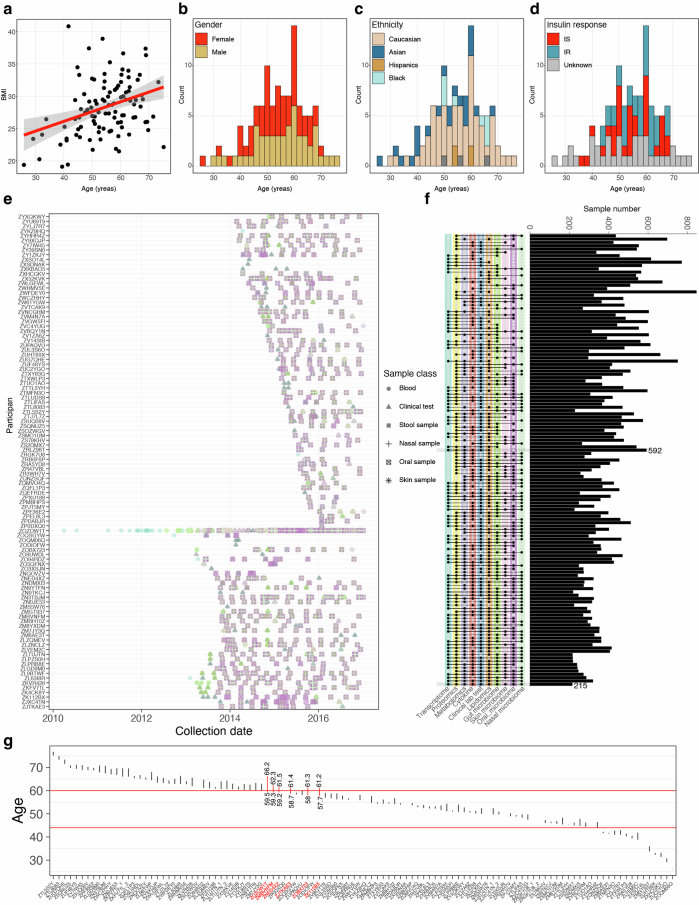
Demographic data of all the participants in the study. **a**, The ages positively correlate with BMI. The shaded area around the regression line represents the 95% confidence interval. **b**, Gender with age. **c**, Ethnicity with age. **d**, Insulin response with age. **e**, biological sample collection for all the participants. **f**, Overlap of the different kinds of omics data. **g**, The age range for each participant in this study.

We collected longitudinal biological samples from 108 participants over several years, with a median tracking period of 1.7 years and a maximum period of 6.8 years, and conducted multi-omics profiling on the samples. The participants were sampled every 3–6 months while healthy and had diverse ethnic backgrounds and ages ranging from 25 years to 75 years (median, 55.7 years). The participants’ body mass index (BMI) ranged from 19.1 kg m^−2^ to 40.8 kg m^−2^ (median, 28.2 kg m^−2^). Among the participants, 51.9% were female (Fig. 1a and Extended Data Fig. 1a–d). For each visit, we collected blood, stool, skin swab, oral swab and nasal swab samples. In total, 5,405 biological samples (including 1,440 blood samples, 926 stool samples, 1,116 skin swab samples, 1,001 oral swab samples and 922 nasal swab samples) were collected. The biological samples were used for multi-omics data acquisition (including transcriptomics from peripheral blood mononuclear cells (PBMCs), proteomics from plasma, metabolomics from plasma, cytokines from plasma, clinical laboratory tests from plasma, lipidomics from plasma, stool microbiome, skin microbiome, oral microbiome and nasal microbiome; Methods). In total, 135,239 biological features (including 10,346 transcripts, 302 proteins, 814 metabolites, 66 cytokines, 51 clinical laboratory tests, 846 lipids, 52,460 gut microbiome taxons, 8,947 skin microbiome taxons, 8,947 oral microbiome taxons and 52,460 nasal microbiome taxons) were acquired, resulting in 246,507,456,400 data points (Fig. 1b and Extended Data Fig. 1e,f). The average sampling period and number of samples for each participant were 626 days and 47 samples, respectively. Notably, one participant was deeply monitored for 6.8 years (2,471 days), during which 367 samples were collected (Fig. 1c). Overall, this extensive and longitudinal multi-omics dataset enables us to examine the molecular changes that occur during the human aging process. The detailed characteristics of all participants are provided in the Supplementary Data. For each participant, the omics data were aggregated and averaged across all healthy samples to represent the individual’s mean value, as detailed in the Methods section. Compared to cross-sectional cohorts, which have only a one-time point sample from each participant, our longitudinal dataset, which includes multiple time point samples from each participant, is more robust for detecting complex aging-related changes in molecules and functions. This is because analysis of multi-time point samples can detect participants’ baseline and robustly evaluate individuals’ longitudinal molecular changes.

**Extended Data Fig. 2:**
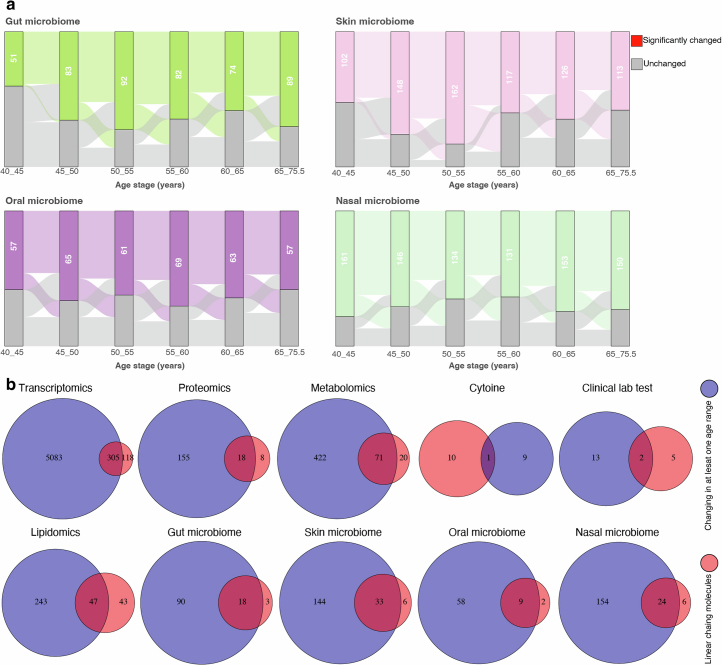
Most of the molecules change nonlinearly during human aging. **a**, Differential expressional microbes in different age ranges compared to baselines (25 – 40 years old, two-sided Wilcoxon test, *p*-value < 0.05). **b**, Most of the linear changing molecules and microbiota are also included in the molecules/microbes that significantly dysregulated at least one age range.

We included samples only from healthy visits and adjusted for confounding factors (for example, BMI, sex, insulin resistance/insulin sensitivity (IRIS) and ethnicity; Extended Data Fig. 1a–d), allowing us to discern the molecules and microbes genuinely associated with aging (Methods). Two common and traditional approaches, linear regression and Spearman correlation, were first used to identify the linear changing molecules during human aging^5^. The linear regression method is commonly used for linear changing molecules. As expected, both approaches have very high consistent results for each type of omics data (Supplementary Fig. 1a). For convenience, the Spearman correlation approach was used in the analysis. Interestingly, only a small portion of all the molecules and microbes (749 out of 11,305, 6.6%; only genus level was used for microbiome data; Methods) linearly changed during human aging (Fig. 1d and Supplementary Fig. 1b), consistent with our previous studies^5^ (Methods). Next, we examined nonlinear effects by categorizing all participants into distinct age stages according to their ages and investigated the dysregulated molecules within each age stage compared to the baseline (25–40 years old; Methods). Interestingly, using this approach, 81.03% of molecules (9,106 out of 11,305) exhibited changes in at least one age stage compared to the baseline (Fig. 1e and Extended Data Fig. 2a). Remarkably, the percentage of linear changing molecules was relatively small compared to the overall dysregulated molecules during aging (mean, 16.2%) (Fig. 1f and Extended Data Fig. 2b). To corroborate our findings, we employed a permutation approach to calculate permutated *P* values, which yielded consistent results (Methods). The heatmap depicting all dysregulated molecules also clearly illustrates pronounced nonlinear changes (Fig. 1g). Taken together, these findings strongly suggest that a substantial number of molecules and microbes undergo nonlinear changes throughout human aging.

### Clustering reveals nonlinear multi-omics changes during aging

**Fig. 2:**
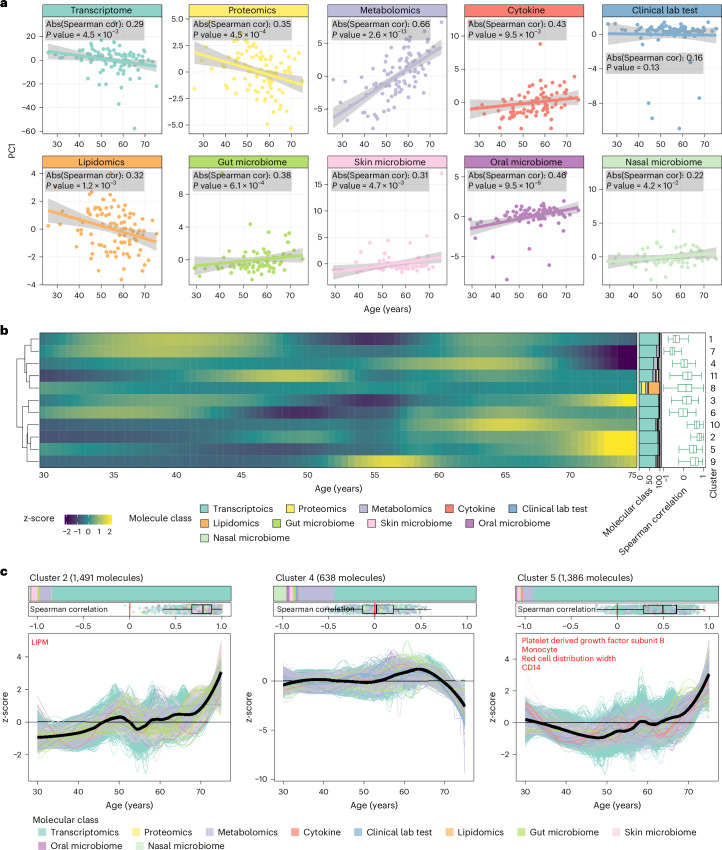
Clustering reveals nonlinear changes in multi-omics profiling during human aging. **a**, Spearman correlation (cor) between the first principal component and ages for each type of omics data. The shaded area around the regression line represents the 95% confidence interval. **b**, The heatmap shows the molecular trajectories in 11 clusters during human aging. The right stacked bar plots show the percentages of different kinds of omics data, and the right box plots show the correlation distribution between features and ages (*n* = 108 participants). **c**, Three notable clusters of molecules that exhibit clear and straightforward nonlinear changes during human aging. The top stacked bar plots show the percentages of different kinds of omics data, and the top box plots show the correlation distribution between features and ages (*n* = 108 participants). The box plot shows the median (line), interquartile range (IQR) (box) and whiskers extending to 1.5 × IQR. Bars and lines are color-coded by omics type. Abs, absolute.

**Fig. 3:**
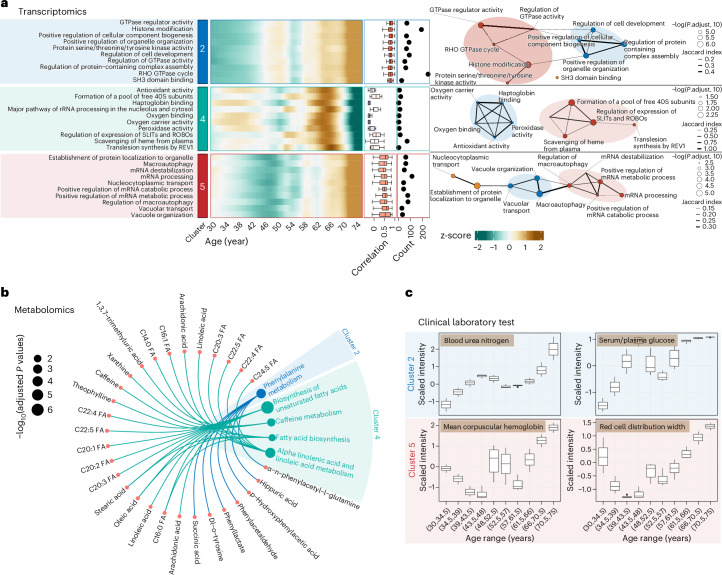
Functional analysis of nonlinear changing molecules in each cluster. **a**, Pathway enrichment and module analysis for each transcriptome cluster. The left panel is the heatmap for the pathways that undergo nonlinear changes across aging. The right panel is the pathway similarity network (Methods) (*n* = 108 participants). **b**, Pathway enrichment for metabolomics in each cluster. Enriched pathways and related metabolites are illustrated (Benjamini–Hochberg-adjusted *P* < 0.05). **c**, Four clinical laboratory tests that change during human aging: blood urea nitrogen, serum/plasma glucose, mean corpuscular hemoglobin and red cell distribution width (*n* = 108 participants). The box plot shows the median (line), interquartile range (IQR) (box) and whiskers extending to 1.5 × IQR.

**Extended Data Fig. 3:**
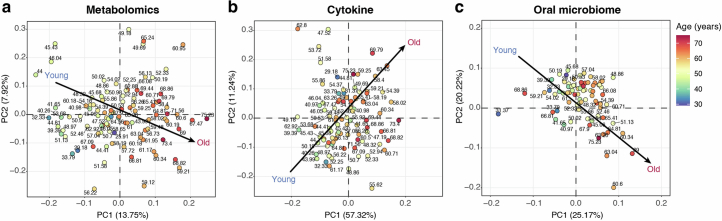
Omics data can represent aging. PCA score plot of metabolomics data (**a**), cytokine (**b**), and oral microbiome (**c**).

Next, we assessed whether the multi-omics data collected from the longitudinal cohort could serve as reliable indicators of the aging process. Our analysis revealed a substantial correlation between a significant proportion of the omics data and the ages of the participants (Fig. 2a). Particularly noteworthy was the observation that, among all the omics data examined, metabolomics, cytokine and oral microbiome data displayed the strongest association with age (Fig. 2a and Extended Data Fig. 3a–c). Partial least squares (PLS) regression was further used to compare the strength of the age effect across different omics data types. The results are consistent with the results presented above in Fig. 2a (Methods). These findings suggest the potential utility of these datasets as indicators of the aging process while acknowledging that further research is needed for validation^4^. As the omics data are not accurately matched across all the samples, we then smoothed the omics data using our previously published approach^19^ (Methods and Supplementary Fig. 2a–c). Next, to reveal the specific patterns of molecules that change during human aging, we then grouped all the molecules with similar trajectories using an unsupervised fuzzy c-means clustering approach^19^ (Methods, Fig. 3b and Supplementary Fig. 2d,e). We identified 11 clusters of molecular trajectories that changed during aging, which ranged in size from 638 to 1,580 molecules/microbes (Supplementary Fig. 2f and Supplementary Data). We found that most molecular patterns exhibit nonlinear changes, indicating that aging is not a linear process (Fig. 2b). Among the 11 identified clusters, three distinct clusters (2, 4 and 5) displayed compelling, straightforward and easily understandable patterns that spanned the entire lifespan (Fig. 2c). Most molecules within these three clusters primarily consist of transcripts (Supplementary Fig. 2f), which is expected because transcripts dominate the multi-omics data (8,556 out of 11,305, 75.7%). Cluster 4 exhibits a relatively stable pattern until approximately 60 years of age, after which it shows a rapid decrease (Fig. 2c). Conversely, clusters 2 and 5 display fluctuations before 60 years of age, followed by a sharp increase and an upper inflection point at approximately 55–60 years of age (Fig. 2c). We also attempted to observe this pattern of molecular change during aging individually. The participant with the longest follow-up period of 6.8 years (Fig. 1c) approached the age of 60 years (range, 59.5–66.3 years; Extended Data Fig. 1g), and it was not possible to identify obvious patterns in this short time window (Supplementary Fig. 2g). Tracking individuals longitudinally over longer periods (decades) will be required to observe these trajectories at an individual level.

**Extended Data Fig. 4:**
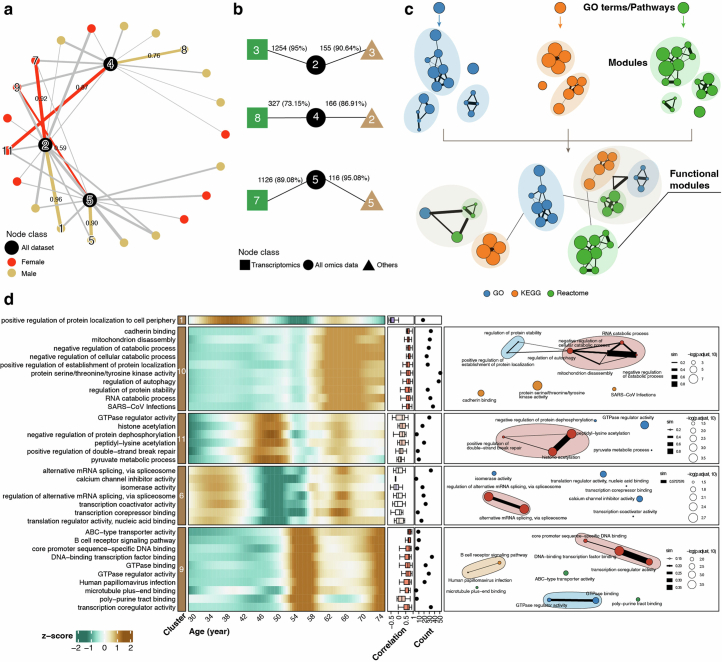
Functional analysis of molecules in different clusters. **a**, The Jaccard index between clusters from different datasets. **b**, The overlap between clusters using different types of omics data. **c**, Functional module detection and identification. **d**, Functional analysis of nonlinear changing molecules for all clusters.

Although confounders, including sex, were corrected before analysis (Methods), we acknowledge that the age range for menopause in females is typically between 45 years and 55 years of age^20^, which is very close to the major transition points in all three clusters (Fig. 2c). Therefore, we conducted further investigation into whether the menopausal status of females in the dataset contributed to the observed transition point at approximately 55 years of age (Fig. 2c) by performing separate clustering analyses on the male and female datasets. Surprisingly, both the male and female datasets exhibited similar clusters, as illustrated in Extended Data Fig. 4a. This suggests that the transition point observed at approximately 55 years of age is not solely attributed to female menopause but, rather, represents a common phenomenon in the aging process of both sexes. This result is consistent with previous studies^14,15^, further supporting the notion that this transition point is a major characteristic feature of human aging. Moreover, to investigate the possibility that the transcriptomics data might skew the results toward transcriptomic changes as age-related factors, we conducted two additional clustering analyses—one focusing solely on transcriptomic data and another excluding it. Interestingly, both analyses yielded nearly identical three-cluster configurations, as observed using the complete omics dataset (Extended Data Fig. 4b). This reinforces the robustness of the identified clusters and confirms that they are consistent across various omics platforms, not just driven by transcriptomic data.

### Nonlinear changes in function and disease risk during aging

To gain further insight into the biological functions associated with the nonlinear changing molecules within the three identified clusters, we conducted separate functional analyses for transcriptomics, proteomics and metabolomics datasets for all three clusters. In brief, we constructed a similarity network using enriched pathways from various databases (Gene Ontology (GO), Kyoto Encyclopedia of Genes and Genomes (KEGG) and Reactome) and identified modules to eliminate redundant annotations. We then used all modules from different databases to reduce redundancy further using the same approach and define the final functional modules (Methods, Extended Data Fig. 4c and Supplementary Data). We identified some functional modules that were reported in previous studies, but we defined their more accurate patterns of change during human aging. Additionally, we also found previously unreported potential functional modules during human aging (Supplementary Data). For instance, in cluster 2, we identified a transcriptomic module associated with GTPase activity (adjusted *P* = 1.64 × 10^−6^) and histone modification (adjusted *P* *=* 6.36 × 10^−7^) (Fig. 3a). Because we lack epigenomic data in this study, our findings should be validated through additional experiments in the future. GTPase activity is closely correlated with programmed cell death (apoptosis), and some previous studies showed that this activity increases during aging^21^. Additionally, histone modifications have been demonstrated to increase during human aging^22^. In cluster 4, we identified one transcriptomics module associated with oxidative stress; this module includes antioxidant activity, oxygen carrier activity, oxygen binding and peroxidase activity (adjusted *P* = 0.029) (Fig. 3a). Previous studies demonstrated that oxidative stress and many reactive oxygen species (ROS) are positively associated with increased inflammation in relation to aging^23^. In cluster 5, the first transcriptomics module is associated with mRNA stability, which includes mRNA destabilization (adjusted *P* *=* 0.0032), mRNA processing (adjusted *P* *=* 3.2 × 10^−4^), positive regulation of the mRNA catabolic process (adjusted *P* *=* 1.46 × 10^−4^) and positive regulation of the mRNA metabolic process (adjusted *P* *=* 0.00177) (Fig. 3a). Previous studies showed that mRNA turnover is associated with aging^24^. The second module is associated with autophagy (Fig. 3a), which increases during human aging, as demonstrated in previous studies^25^.

In addition, we also identified certain modules in the clusters that suggest a nonlinear increase in several disease risks during human aging. For instance, in cluster 2, where components increase gradually and then rapidly after age 60, the phenylalanine metabolism pathway (adjusted *P* *=* 4.95 × 10^−4^) was identified (Fig. 3b). Previous studies showed that aging is associated with a progressive increase in plasma phenylalanine levels concomitant with cardiac dysfunction, and dysregulated phenylalanine catabolism is a factor that triggers deviations from healthy cardiac aging trajectories^26^. Additionally, C-X-C motif chemokine 5 (CXCL5 or ENA78) from proteomics data, which has higher concentrations in atherosclerosis^27^, is also detected in cluster 2 (Supplementary Data). The clinical laboratory test blood urea nitrogen, which provides important information about kidney function, is also detected in cluster 2 (Fig. 3c). This indicates that kidney function nonlinearly decreases during aging. Furthermore, the clinical laboratory test for serum/plasma glucose, a marker of type 2 diabetes (T2D), falls within cluster 2. This is consistent with and supported by many previous studies demonstrating that aging is a major risk factor for T2D^28^. Collectively, these findings suggest a nonlinear escalation in the risk of cardiovascular and kidney diseases and T2D with advancing age, particularly after the age of 60 years (Fig. 2c).

The identified modules in cluster 4 also indicate a nonlinear increase in disease risks. For instance, the unsaturated fatty acids biosynthesis pathway (adjusted *P* *=* 4.71 × 10^−7^) is decreased in cluster 4. Studies have shown that unsaturated fatty acids are helpful in reducing CVD risk and maintaining brain function^29,30^. The pathway of alpha-linolenic acid and linolenic acid metabolism (adjusted *P* *=* 1.32 × 10^−4^) can reduce aging-associated diseases, such as CVD^31^. We also detected the caffeine metabolism pathway (adjusted *P* *=* 7.34 × 10^−5^) in cluster 4, which suggests that the ability to metabolize caffeine decreases during aging. Additionally, the cytokine MCP1 (chemokine (C-C motif) ligand 2 (CCL2)), a member of the CC chemokine family, plays an important immune regulatory role and is also in cluster 4 (Supplementary Data). These findings further support previous observations and highlight the nonlinear increase in age-related disease risk as individuals age.

Cluster 5 comprises the clinical tests of mean corpuscular hemoglobin and red cell distribution width (Fig. 3c). These tests assess the average hemoglobin content per red blood cell and the variability in the size and volume of red blood cells, respectively. These findings align with the aforementioned transcriptomic data, which suggest a nonlinear reduction in the oxygen-carrying capacity associated with the aging process.

Aside from these three distinct clusters (Fig. 2c), we also conducted pathway enrichment analysis across all other eight clusters, which displayed highly nonlinear trajectories, employing the same method (Fig. 2b and Supplementary Data). Notably, cluster 11 exhibited a consistent increase up until the age of 50, followed by a decline until the age of 56, after which no substantial changes were observed up to the age of 75. A particular transcriptomics module related to DNA repair was identified, encompassing three pathways: positive regulation of double-strand break repair (adjusted *P* *=* 0.042), peptidyl−lysine acetylation (adjusted *P* *=* 1.36 × 10^−5^) and histone acetylation (adjusted *P* *=* 3.45 × 10^−4^) (Extended Data Fig. 4d). These three pathways are critical in genomic stability, gene expression and metabolic balances, with their levels diminishing across the human lifespan^32–34^. Our findings reveal a nonlinear alteration across the human lifespan in these pathways, indicating an enhancement in DNA repair capabilities before the age of 50, a marked reduction between the ages of 50 and 56 and stabilization after that until the age of 75. The pathway enrichment results for all clusters are detailed in the Supplementary Data.

Altogether, the comprehensive functional analysis offers valuable insights into the nonlinear changes observed in molecular profiles and their correlations with biological functions and disease risks across the human lifespan. Our findings reveal that individuals aged 60 and older exhibit increased susceptibility to CVD, kidney issues and T2D. These results carry important implications for both the diagnosis and prevention of these diseases. Notably, many clinically actionable markers were identified, which have the potential for improved healthcare management and enhanced overall well-being of the aging population.

### Uncovering waves of aging-related molecules during aging

**Fig. 4:**
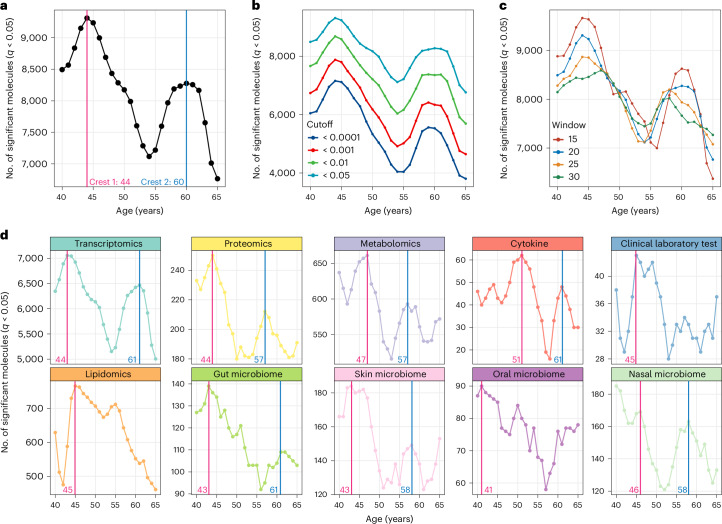
Waves of molecules and microbes during aging. **a**, Number of molecules and microbes differentially expressed during aging. Two local crests at the ages of 44 years and 60 years were identified. **b**,**c**, The same waves were detected using different *q* value (**b**) and window (**c**) cutoffs. **d**, The number of molecules/microbes differentially expressed for different types of omics data during human aging.

Although the trajectory clustering approaches described above effectively identify nonlinear changing molecules and microbes that exhibit clear and compelling patterns throughout human aging, it may not be as effective in capturing substantial changes that occur at specific chronological aging periods. In such cases, alternative analytical approaches may be necessary to detect and characterize these dynamics. To gain a comprehensive understanding of changes in multi-omics profiling during human aging, we used a modified version of the DE-SWAN algorithm^14^, as described in the Methods section. This algorithm identifies dysregulated molecules and microbes throughout the human lifespan by analyzing molecule levels within 20-year windows and comparing two groups in 10-year parcels while sliding the window incrementally from young to old ages^14^. Using this approach and multiomics data, we detected changes at specific stages of lifespan and uncovered the sequential effects of aging. Our analysis revealed thousands of molecules exhibiting changing patterns throughout aging, forming distinct waves, as illustrated in Fig. 3a. Notably, we observed two prominent crests occurring around the ages of 45 and 65, respectively (Fig. 4a). Notably, too, these crests were consistent with findings from a previous study that included only proteomics data^14^. Specifically, crest 2 aligns with our previous trajectory clustering result, indicating a turning point at approximately 60 years of age (Fig. 2c).

To demonstrate the significance of the two crests, we employed different *q* value cutoffs and sliding window parameters, which consistently revealed the same detectable waves (Fig. 4b,c and Supplementary Fig. 4a,b). Furthermore, when we permuted the ages of individuals, the crests disappeared (Supplementary Figs. 3a and 4c) (Methods). These observations highlight the robustness of the two major waves of aging-related molecular changes across the human lifespan. Although we already accounted for confounders before our statistical analysis, we took additional steps to explore their impact. Specifically, we investigated whether confounders, such as insulin sensitivity, sex and ethnicity, differed between the two crests across various age ranges. As anticipated, these confounders did not show significant differences across other age brackets (Supplementary Fig. 4d). This further supports our conclusion that the observed differences in the two crests are attributable to age rather than other confounding variables.

The identified crests represent notable milestones in the aging process and suggest specific age ranges where substantial molecular alterations occur. Therefore, we investigated the age-related waves for each type of omics data. Interestingly, most types of omics data exhibited two distinct crests that were highly robust (Fig. 3b and Supplementary Fig. 4). Notably, the proteomics data displayed two age-related crests at ages around 40 years and 60 years. Only a small overlap was observed between our dataset and the results from the previous study (1,305 proteins versus 302 proteins, with only 75 proteins overlapping). The observed pattern in our study was largely consistent with the previous findings^14^. However, our finding that many types of omics data, including transcriptomics, proteomics, metabolomics, cytokine, gut microbiome, skin microbiome and nasal microbiome, exhibit these waves, often with a similar pattern as the proteomics data (Fig. 4d), supports the hypothesis that aging-related changes are not limited to a specific omics layer but, rather, involve a coordinated and systemic alteration across multiple molecular components. Identifying consistent crests across different omics data underscores the robustness and reliability of these molecular milestones in the aging process.

**Fig. 5:**
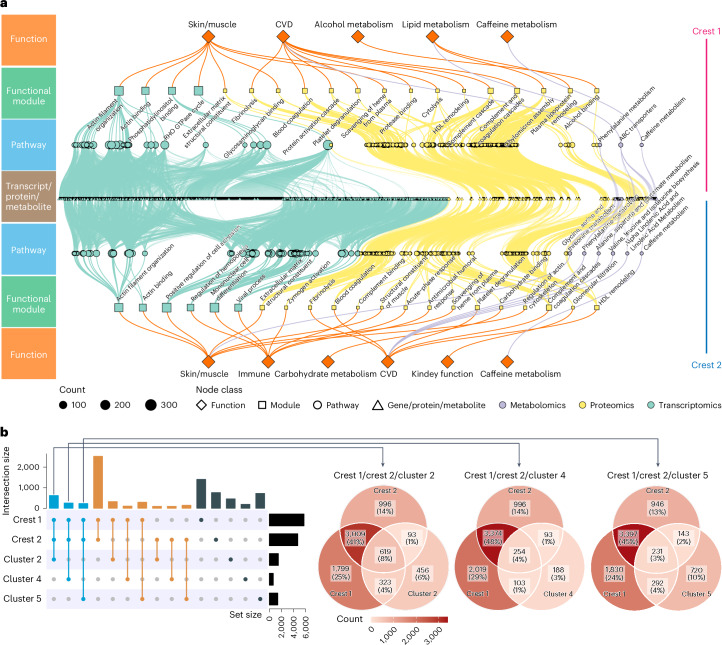
Functional analysis of aging-related waves of molecules across the human lifespan. **a**, Pathway enrichment and biological functional module analysis for crests 1 and 2. Dots and lines are color-coded by omics type. **b**, The overlapping of molecules between two crests and three clusters.

**Extended Data Fig. 5:**
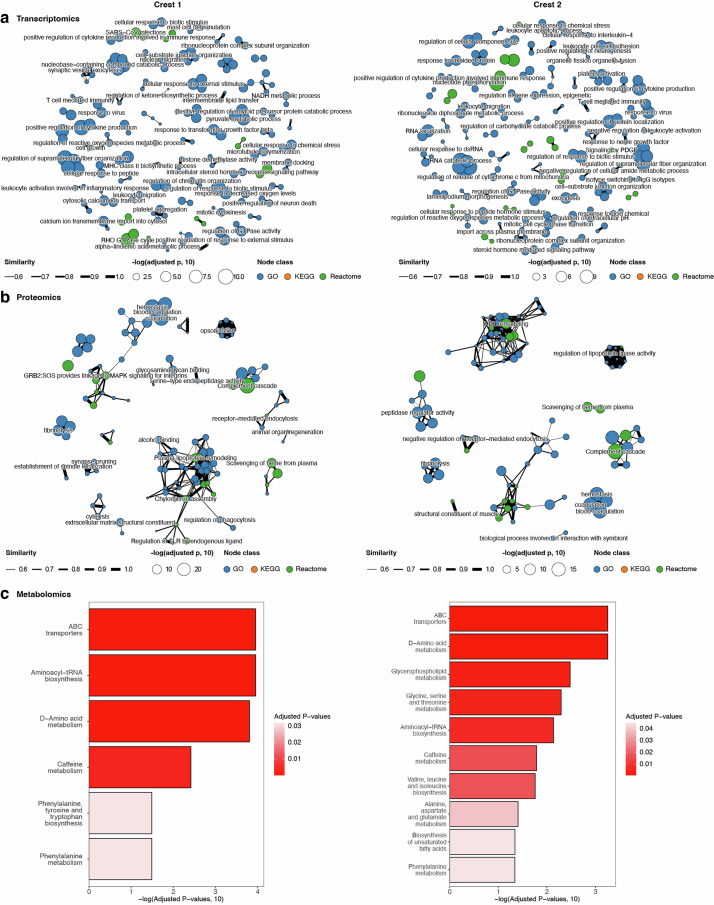
Function annotation for significantly dysregulated molecules in crest 1 and 2. **a**, Transcriptomics data. **b**, Proteomics data. **c**, Metabolomics data.

**Extended Data Fig. 6:**
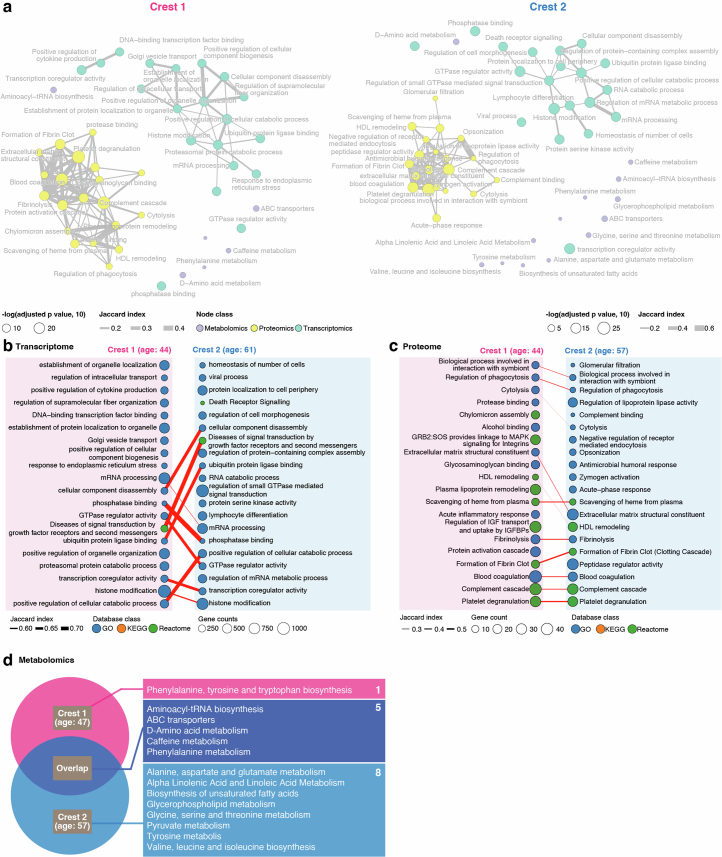
Pathways enrichment results for crest 1 and 2. **a**, The final functional modules identified for Crest 1 and 2. **b**, The pathway enrichment analysis results for transcriptomics data. **c**, The pathway enrichment analysis results for proteomics data. **d**, The pathway enrichment results for metabolomics data.

Next, we investigated the roles and functions of dysregulated molecules within two distinct crests. Notably, we found that the two crests related to aging predominantly consisted of the same molecules (Supplementary Fig. 6). To focus on the unique biological functions associated with each crest and eliminate commonly occurring molecules, we removed background molecules present in most stages. To explore the specific biological functions associated with each type of omics data (transcriptomics, proteomics and metabolomics) for both crests, we employed the function annotation approach described above (Methods). In brief, we constructed a similarity network of enriched pathways and identified modules to remove redundant annotations (Supplementary Fig. 6 and Extended Data Fig. 5a,b). Furthermore, we applied the same approach to all modules to reduce redundancy and identify the final functional modules (Methods and Extended Data Fig. 6a). Our analysis revealed significant changes in multiple modules associated with the two crests (Extended Data Fig. 6b–d). To present the results clearly, Fig. 5a displays the top 20 pathways (according to adjusted *P* value) for each type of omics data, and the Supplementary Data provides a comprehensive list of all identified functional modules.

Interestingly, the analysis identifies many dysregulated functional modules in crests 1 and 2, indicating a nonlinear risk for aging-related diseases. In particular, several modules associated with CVD were identified in both crest 1 and crest 2 (Fig. 5a), which is consistent with the above results (Fig. 3b). For instance, the dysregulation of platelet degranulation (crest 1: adjusted *P* *=* 1.77 × 10^−30^; crest 2: adjusted *P* *=* 1.73 × 10^−26^)^35,36^, complement cascade (crest 1: adjusted *P* *=* 3.84 × 10^−30^; crest 2: adjusted *P* *=* 2.02 × 10^−28^), complement and coagulation cascades (crest 1: adjusted *P* *=* 1.78 × 10^−46^; crest 2: adjusted *P* *=* 2.02 × 10^−28^)^37,38^, protein activation cascade (crest 1: adjusted *P* *=* 1.56 × 10^−17^; crest 2: adjusted *P* *=* 1.61 × 10^−8^) and protease binding (crest 1: adjusted *P* *=* 2.7 × 10^−6^; crest 2: adjusted *P* *=* 0.0114)^39^ have various effects on the cardiovascular system and can contribute to various CVDs. Furthermore, blood coagulation (crest 1: adjusted *P* *=* 1.48 × 10^−28^; crest 2: adjusted *P* *=* 9.10 × 10^−17^) and fibrinolysis (crest 1: adjusted *P* *=* 2.11 × 10^−15^; crest 2: adjusted *P* *=* 1.64 × 10^−10^) were also identified, which are essential processes for maintaining blood fluidity, and dysregulation in these modules can lead to thrombotic and cardiovascular events^40,41^. We also identified certain dysregulated metabolic modules associated with CVD. For example, aging has been linked to an incremental rise in plasma phenylalanine levels (crest 1: adjusted *P* *=* 9.214 × 10^−4^; crest 2: adjusted *P* *=* 0.0453), which can contribute to the development of cardiac hypertrophy, fibrosis and dysfunction^26^. Branched-chain amino acids (BCAAs), including valine, leucine and isoleucine (crest 1: adjusted *P*: not significant (NS); crest 2: adjusted *P* *=* 0.0173), have also been implicated in CVD development^42,43^ and T2D, highlighting their relevance in CVD pathophysiology. Furthermore, research suggests that alpha-linolenic acid (ALA) and linoleic acid metabolism (crest 1: adjusted *P*: NS; crest 2: adjusted *P* *=* 0.0217) may be protective against coronary heart disease^44,45^. Our investigation also identified lipid metabolism modules that are associated with CVD, including high-density lipoprotein (HDL) remodeling (crest 1: adjusted *P* *=* 1.073 × 10^−8^; crest 2: adjusted *P* *=* 2.589 × 10^−9^) and glycerophospholipid metabolism (crest 1: adjusted *P*: NS; crest 2: adjusted *P* *=* 0.0033), which influence various CVDs^46–48^.

In addition, the dysregulation of skin and muscle stability was found to be increased at crest 1 and crest 2, as evidenced by the identification of numerous modules associated with these processes (Fig. 5a,b). This suggests that the aging of skin and muscle is markedly accelerated at crest 1 and crest 2. The extracellular matrix (ECM) provides structural stability, mechanical strength, elasticity and hydration to the tissues and cells, and the ECM of the skin is mainly composed of collagen, elastin and glycosaminoglycans (GAGs)^49^. Phosphatidylinositols are a class of phospholipids that have various roles in cytoskeleton organization^50^. Notably, the dysregulation of ECM structural constituent (crest 1: adjusted *P* *=* 3.32 × 10^−8^; crest 2: adjusted *P* *=* 1.61 × 10^−8^), GAG binding (crest 1: adjusted *P* *=* 1.805 × 10^−8^; crest 2: adjusted *P* *=* 4.093 × 10^−6^) and phosphatidylinositol binding (crest 1: adjusted *P* *=* 3.391 × 10^−6^; crest 2: adjusted *P* *=* 7.832 × 10^−6^) were identified^51,52^. We also identified cytolysis (crest 1: adjusted *P* *=* 2.973 × 10^−5^; crest 2: adjusted *P*: NS), which can increase water loss^53^. The dysregulated actin binding (crest 1: adjusted *P* *=* 3.536 × 10^−8^; crest 2: adjusted *P* *=* 3.435 × 10^−9^), actin filament organization (crest 1: adjusted *P* *=* 8.406 × 10^−9^; crest 2: adjusted *P* *=* 1.157 × 10^−9^) and regulation of actin cytoskeleton (crest 1: adjusted *P* *=* 0.00090242; crest 2: adjusted *P* *=* 6.788 × 10^−4^) were identified, which affect the structure and function of various tissues^54–58^. Additionally, cell adhesion is the attachment of a cell to another cell or to ECM via adhesion molecules^59^. We identified the positive regulation of cell adhesion (crest 1: adjusted *P* *=* 3.618 × 10^−5^; crest 2: adjusted *P* *=* 8.272 × 10^−9^) module, which can prevent or delay skin aging^60,61^. Threonine can affect sialic acid production, which is involved in cell adhesion^62^. We also identified the glycine, serine and threonine metabolism (crest 1: adjusted *P*: NS; crest 2: adjusted *P* *=* 0.00506)^62^. Additionally, scavenging of heme from plasma was identified (crest 1: adjusted *P* *=* 1.176 × 10^−11^; crest 2: adjusted *P* *=* 1.694 × 10^−8^), which can modulate skin aging as excess-free heme can damage cellular components^63,64^. Rho GTPases regulate a wide range of cellular responses, including changes to the cytoskeleton and cell adhesion (RHO GTPase cycle, crest 1: adjusted *P* *=* 9.956 × 10^−10^; crest 2: adjusted *P* *=* 1.546 × 10^−5^)^65^. In relation to muscle, previous studies demonstrated that muscle mass decreases by approximately 3–8% per decade after the age of 30, with an even higher decline rate after the age of 60, which consistently coincides with the observed second crest^66^. Interestingly, we identified dysregulation in the module associated with the structural constituent of muscle (crest 1: adjusted *P* *=* 0.00565; crest 2: adjusted *P* *=* 0.0162), consistent with previous findings^66^. Furthermore, we identified the pathway associated with caffeine metabolism (crest 1: adjusted *P* *=* 0.00378; crest 2: adjusted *P* *=* 0.0162), which is consistent with our observations above (Fig. 2b) and implies that the capacity to metabolize caffeine undergoes a notable alteration not only around 60 years of age but also around the age of 40 years.

In crest 1, we identified specific modules associated with lipid and alcohol metabolism. Previous studies established that lipid metabolism declines with human aging^67^. Our analysis revealed several modules related to lipid metabolism, including plasma lipoprotein remodeling (crest 1: adjusted *P* *=* 2.269 × 10^−9^), chylomicron assembly (crest 1: adjusted *P* *=* 9.065 × 10^−7^) and ATP-binding cassette (ABC) transporters (adjusted *P* *=* 1.102 × 10^−4^). Moreover, we discovered a module linked to alcohol metabolism (alcohol binding, adjusted *P* *=* 8.485 × 10^−7^), suggesting a decline in alcohol metabolization efficiency with advancing age, particularly around the age of 40, when it significantly diminishes. In crest 2, we observed prominent modules related to immune dysfunction, encompassing acute-phase response (adjusted *P* *=* 2.851 × 10^−8^), antimicrobial humoral response (adjusted *P* *=* 2.181 × 10^−5^), zymogen activation (adjusted *P* *=* 4.367 × 10^−6^), complement binding (adjusted *P* *=* 0.002568), mononuclear cell differentiation (adjusted *P* *=* 9.352 × 10^−8^), viral process (adjusted *P* *=* 5.124 × 10^−7^) and regulation of hemopoiesis (adjusted *P* *=* 3.522 × 10^−7^) (Fig. 5a). Age-related changes in the immune system, collectively known as immunosenescence, have been extensively documented^68–70^, and our results demonstrate a rapid decline at age 60. Furthermore, we also identified modules associated with kidney function (glomerular filtration, adjusted *P* *=* 0.00869) and carbohydrate metabolism (carbohydrate binding, adjusted *P* *=* 0.01045). Our previous findings indicated a decline in kidney function around the age of 60 years (Fig. 3c), as did the present result of this observation. Previous studies indicated the influence of carbohydrates on aging, characterized by the progressive decline of physiological functions and increased susceptibility to diseases over time^71,72^.

In summary, our analysis identifies many dysregulated functional modules identified in both crest 1 and crest 2 that underlie the risk for various diseases and alterations of biological functions. Notably, we observed an overlap of dysregulated functional modules among clusters 2, 4 and 6 because they overlap at the molecular level, as depicted in Fig. 5b. This indicates that certain molecular components are shared among these clusters and the identified crests. However, it is important to note that numerous molecules are specific to each of the two approaches employed in our study. This suggests that these two approaches complement each other in identifying nonlinear changes in molecules and functions during human aging. By using both approaches, we were able to capture a more comprehensive understanding of the molecular alterations associated with aging and their potential implications for diseases.

## Discussion

Analyzing a longitudinal multi-omics dataset involving 108 participants, we successfully captured the dynamic and nonlinear molecular changes that occur during human aging. Our study’s strength lies in the comprehensive nature of the dataset, which includes multiple time point samples for each participant. This longitudinal design enhances the reliability and robustness of our findings compared to cross-sectional studies with only one time point sample for each participant. The first particularly intriguing finding from our analysis is that only a small fraction of molecules (6.6%) displayed linear changes throughout human aging (Fig. 1d). This observation is consistent with previous research and underscores the limitations of relying solely on linear regression to understand the complexity of aging-related molecular changes^5^. Instead, our study revealed that a considerable number of molecules (81.0%) exhibited nonlinear patterns (Fig. 1e). Notably, this nonlinear trend was observed across all types of omics data with remarkably high consistency (Fig. 1e,g), highlighting the widespread functionally relevant nature of these dynamic changes. By unveiling the nonlinear molecular alterations associated with aging, our research contributes to a more comprehensive understanding of the aging process and its molecular underpinnings.

To further investigate the nonlinear changing molecules observed in our study, we employed a trajectory clustering approach to group molecules with similar temporal patterns. This analysis revealed the presence of three distinct clusters (Fig. 2c) that exhibited clear and compelling patterns across the human lifespan. These clusters suggest that there are specific age ranges, such as around 60 years old, where distinct and extensive molecular changes occur (Fig. 2c). Functional analysis revealed several modules that exhibited nonlinear changes during human aging. For example, we identified a module associated with oxidative stress, which is consistent with previous studies linking oxidative stress to the aging process^23^ (Fig. 3a). Our analysis indicates that this pathway increases significantly after the age of 60 years. In cluster 5, we identified a transcriptomics module associated with mRNA stabilization and autophagy (Fig. 3a). Both of these processes have been implicated in the aging process and are involved in maintaining cellular homeostasis and removing damaged components. Furthermore, our analysis uncovered nonlinear changes in disease risk across aging. In cluster 2, we identified the phenylalanine metabolism pathway (Fig. 3b), which has been associated with cardiac dysfunction during aging^26^. Additionally, we found that the clinical laboratory tests blood urea nitrogen and serum/plasma glucose increase significantly with age (cluster 2; Fig. 3c), indicating a nonlinear decline in kidney function and an increased risk of T2D with age, with a critical threshold occurring approximately at the age of 60 years. In cluster 4, we identified pathways related to cardiovascular health, such as the biosynthesis of unsaturated fatty acids and caffeine metabolism (Fig. 3b). Overall, our study provides compelling evidence for the existence of nonlinear changes in molecular profiles during human aging. By elucidating the specific functional modules and disease-related pathways that exhibit such nonlinear changes, we contribute to a better understanding of the complex molecular dynamics underlying the aging process and its implications for disease risk.

Although the trajectory clustering approach proves effective in identifying molecules that undergo nonlinear changes, it may not be as proficient in capturing substantial alterations that occur at specific time points without exhibiting a consistent pattern in other stages. We then employed a modified version of the DE-SWAN algorithm^14^ to comprehensively investigate changes in multi-omics profiling throughout human aging. This approach enabled us to identify waves of dysregulated molecules and microbes across the human lifespan. Our analysis revealed two prominent crests occurring around the ages of 40 years and 60 years, which were consistent across various omics data types, suggesting their universal nature (Fig. 4a,e). Notably, in the proteomics data, we observed crests around the ages of 40 years and 60 years, which aligns approximately with a previous study (which reported crests at ages 34 years, 60 years and 78 years)^14^. Due to the age range of our cohort being 25–75 years, we did not detect the third peak. Furthermore, the differences in proteomics data acquisition platforms (mass spectrometry versus SomaScan)^14,73^ resulted in different identified proteins, with only a small overlap (1,305 proteins versus 302 proteins, of which only 75 were shared). This discrepancy may explain the age variation of the first crest identified in the two studies (approximately 10 years). However, despite the differences in the two proteomics datasets, the wave patterns observed in both studies were highly similar^14^ (Fig. 4a). Remarkably, by considering multiple omics data types, we consistently identified similar crests for each type, indicating the universality of these waves of change across plasma molecules and microbes from various body sites (Fig. 4e and Supplementary Fig. 3).

The analysis of molecular functionality in the two distinct crests revealed the presence of several modules, indicating a nonlinear increase in the risks of various diseases (Fig. 5a). Both crest 1 and crest 2 exhibit the identification of multiple modules associated with CVD, which aligns with the aforementioned findings (Fig. 3b). Moreover, we observed an escalated dysregulation in skin and muscle functioning in both crest 1 and crest 2. Additionally, we identified a pathway linked to caffeine metabolism, indicating a noticeable alteration in caffeine metabolization not only around the age of 60 but also around the age of 40. This shift may be due to either a metabolic shift or a change in caffeine consumption. In crest 1, we also identified specific modules associated with lipid and alcohol metabolism, whereas crest 2 demonstrated prominent modules related to immune dysfunction. Furthermore, we also detected modules associated with kidney function and carbohydrate metabolism, which is consistent with our above results. These findings reinforce our previous observations regarding a decline in kidney function around the age of 60 years (Fig. 3c) while shedding light on the impact of dysregulated functional modules in both crest 1 and crest 2, suggesting nonlinear changes in disease risk and functional dysregulation. Notably, we identified an overlap of dysregulated functional modules among clusters 2, 4 and 6, indicating molecular-level similarities between these clusters and the identified crests (Fig. 5b). This suggests the presence of shared molecular components among these clusters and crests. However, it is crucial to note that there are also numerous molecules specific to each of the two approaches employed in our study, indicating that these approaches complement each other in identifying nonlinear changes in molecules and functions during human aging.

The present research is subject to certain constraints. We accounted for many basic characteristics (confounders) of participants in the cohort; but because this study primarily reflects between-individual differences, there may be additional confounders due to the different age distributions of the participants. For example, we identified a notable decrease in oxygen carrier activity around age 60 (Figs. 2c and 3a) and marked variations in alcohol and caffeine metabolism around ages 40 and 60 (Fig. 3a). However, these findings might be shaped by participants’ lifestyle—that is, physical activity and their alcohol and caffeine intake. Regrettably, we do not have such detailed behavioral data for the entire group, necessitating validation in upcoming research. Although initial BMI and insulin sensitivity measurements were available at cohort entry, subsequent metrics during the observation span were absent, marking a study limitation.

A further constraint is our cohort’s modest size, encompassing merely 108 individuals (eight individuals between 25 years and 40 years of age), which hampers the full utilization of deep learning and may affect the robustness of the identification of nonlinear changing features in Fig. 1e. Although advanced computational techniques, including deep learning, are pivotal for probing nonlinear patterns, our sample size poses restrictions. Expanding the cohort size in subsequent research would be instrumental in harnessing the full potential of machine learning tools. Another limitation of our study is that the recruitment of participants was within the community around Stanford University, driven by rigorous sample collection procedures and the substantial expenses associated with setting up a longitudinal cohort. Although our participants exhibited a considerable degree of ethnic age and biological sex diversity (Fig. 1a and Supplementary Data), it is important to acknowledge that our cohort may not fully represent the diversity of the broader population. The selectivity of our cohort limits the generalizability of our findings. Future studies should aim to include a more diverse cohort to enhance the external validity and applicability of the results.

In addition, the mean observation span for participants was 626 days, which is insufficient for detailed inflection point analyses. Our cohort’s age range of 25–70 years lacks individuals who lie outside of this range. The molecular nonlinearity detected might be subject to inherent variations or oscillations, a factor to consider during interpretation. Our analysis has not delved into the nuances of the dynamical systems theory, which provides a robust mathematical framework for understanding observed behaviors. Delving into this theory in future endeavors may yield enhanced clarity and interpretation of the data.

Moreover, it should be noted that, in our study, the observed nonlinear molecular changes occurred across individuals of varying ages rather than within the same individuals. This is attributed to the fact that, despite our longitudinal study, the follow-up period for our participants was relatively brief for following aging patterns (median, 1.7 years; Extended Data Fig. 1g). Such a timeframe is inadequate for detecting nonlinear molecular changes that unfold over decades throughout the human lifespan. Addressing this limitation in future research is essential.

Lastly, our study’s molecular data are derived exclusively from blood samples, casting doubt on its direct relevance to specific tissues, such as the skin or muscles. We propose that blood gene expression variations might hint at overarching physiological alterations, potentially impacting the ECM in tissues, including skin and muscle. Notably, some blood-based biomarkers and transcripts have demonstrated correlations with tissue modifications, inflammation and other elements influencing the ECM across diverse tissues^74,75^.

In our future endeavors, the definitive confirmation of our findings hinges on determining if nonlinear molecular patterns align with nonlinear changes in functional capacities, disease occurrences and mortality hazards. For a holistic grasp of this, amalgamating multifaceted data from long-term cohort studies covering several decades becomes crucial. Such data should encompass molecular markers, comprehensive medical records, functional assessments and mortality data. Moreover, employing cutting-edge statistical techniques is vital to intricately decipher the ties between these nonlinear molecular paths and health-centric results.

In summary, the unique contribution of our study lies not merely in reaffirming the nonlinear nature of aging but also in the depth and breadth of the multi-omics data that we analyzed. Our study goes beyond stating that aging is nonlinear by identifying specific patterns, inflection points and potential waves in aging across multiple layers of biological data during human aging. Identifying specific clusters with distinct patterns, functional implications and disease risks enhances our understanding of the aging process. By considering the nonlinear dynamics of aging-related changes, we can gain insights into specific periods of significant changes (around age 40 and age 60) and the molecular mechanisms underlying age-related diseases, which could lead to the development of early diagnosis and prevention strategies. These comprehensive multi-omics data and the approach allow for a more nuanced understanding of the complexities involved in the aging process, which we think adds value to the existing body of research. However, further research is needed to validate and expand upon these findings, potentially incorporating larger cohorts to capture the full complexity of aging.

## Methods

The participant recruitment, sample collection, data acquisition and data processing were documented in previous studies conducted by Zhou et al.^76^, Ahadi et al.^5^, Schüssler-Fiorenza Rose et al.^77^, Hornburg et al.^78^ and Zhou et al.^79^.

### Participant recruitment

Participants provided informed written consent for the study under research protocol 23602, which was approved by the Stanford University institutional review board. This study adheres to all relevant ethical regulations, ensuring informed consents were obtained from all participants. All participants consented to publication of potentially identifiable information. The cohort comprised 108 participants who underwent follow-up assessments. Exclusion criteria encompassed conditions such as anemia, kidney disease, a history of CVD, cancer, chronic inflammation or psychiatric illnesses as well as any prior bariatric surgery or liposuction. Each participant who met the eligibility criteria and provided informed consent underwent a one-time modified insulin suppression test to quantify insulin-mediated glucose uptake at the beginning of the enrollment^76^. The steady-state plasma glucose (SSPG) levels served as a direct indicator of each individual’s insulin sensitivity in processing a glucose load. We categorized individuals with SSPG levels below 150 mg dl^−1^ as insulin sensitive and those with levels of 150 mg dl^−1^ or higher as insulin resistant^80,81^. Thirty-eight participants were missing SSPG values, rendering their insulin resistance or sensitivity status undetermined. We also collected fasting plasma glucose (FPG) data for 69 participants at enrollment. Based on the FPG levels, two participants were identified as having diabetes at enrollment, with FPG levels exceeding 126 mg dl^−1^ (Supplementary Data). Additionally, we measured hemoglobin A1C (HbA1C) levels during each visit, using it as a marker for average glucose levels over the past 3 months: 6.5% or higher indicates diabetes. Accordingly, four participants developed diabetes during the study period. At the beginning of the enrollment, BMI was also measured for each participant. Participants received no compensation.

Comprehensive sample collection was conducted during the follow-up period, and multi-omics data were acquired (Fig. 1b). For each visit, the participants self-reported as healthy or non-healthy^76^. To ensure accuracy and minimize the impact of confounding factors, only samples from individuals classified as healthy were selected for subsequent analysis.

### Transcriptomics

Transcriptomic profiling was conducted on flash-frozen PBMCs. RNA isolation was performed using a QIAGEN All Prep kit. Subsequently, RNA libraries were assembled using an input of 500 ng of total RNA. In brief, ribosomal RNA (rRNA) was selectively eliminated from the total RNA pool, followed by purification and fragmentation. Reverse transcription was carried out using a random primer outfitted with an Illumina-specific adaptor to yield a cDNA library. A terminal tagging procedure was used to incorporate a second adaptor sequence. The final cDNA library underwent amplification. RNA sequencing libraries underwent sequencing on an Illumina HiSeq 2000 platform. Library quantification was performed via an Agilent Bioanalyzer and Qubit fluorometric quantification (Thermo Fisher Scientific) using a high-sensitivity dsDNA kit. After normalization, barcoded libraries were pooled at equimolar ratios into a multiplexed sequencing library. An average of 5–6 libraries were processed per HiSeq 2000 lane. Standard Illumina pipelines were employed for image analysis and base calling. Read alignment to the hg19 reference genome and personal exomes was achieved using the TopHat package, followed by transcript assembly and expression quantification via HTseq and DESeq2. In the realm of data pre-processing, genes with an average read count across all samples lower than 0.5 were excluded. Samples exhibiting an average read count lower than 0.5 across all remaining genes were likewise removed. For subsequent global variance and correlation assessments, genes with an average read count of less than 1 were eliminated.

### Proteomics

Plasma sample tryptic peptides were fractionated using a NanoLC 425 System (SCIEX) operating at a flow rate of 5 μl min^−1^ under a trap-elute configuration with a 0.5 × 10 mm ChromXP column (SCIEX). The liquid chromatography gradient was programmed for a 43-min run, transitioning from 4% to 32% of mobile phase B, with an overall run time of 1 h. Mobile phase A consisted of water with 0.1% formic acid, and mobile phase B was formulated with 100% acetonitrile and 0.1% formic acid. An 8-μg aliquot of non-depleted plasma was loaded onto a 15-cm ChromXP column. Mass spectrometry analysis was executed employing SWATH acquisition on a TripleTOF 6600 system. A set of 100 variable Q1 window SWATH acquisition methods was designed in high-sensitivity tandem mass spectrometry (MS/MS) mode. Subsequent data analysis included statistical scoring of peak groups from individual runs via pyProphet^82^, followed by multi-run alignment through TRIC60, ultimately generating a finalized data matrix with a false discovery rate (FDR) of 1% at the peptide level and 10% at the protein level. Protein quantitation was based on the sum of the three most abundant peptide signals for each protein. Batch effect normalization was achieved by subtracting principal components that primarily exhibited batch-associated variation, using Perseus software v.1.4.2.40.

### Untargeted metabolomics

A ternary solvent system of acetone, acetonitrile and methanol in a 1:1:1 ratio was used for metabolite extraction. The extracted metabolites were dried under a nitrogen atmosphere and reconstituted in a 1:1 methanol:water mixture before analysis. Metabolite profiles were generated using both hydrophilic interaction chromatography (HILIC) and reverse-phase liquid chromatography (RPLC) under positive and negative ion modes. Thermo Q Exactive Plus mass spectrometers were employed for HILIC and RPLC analyses, respectively, in full MS scan mode. MS/MS data were acquired using quality control (QC) samples. For the HILIC separations, a ZIC-HILIC column was used with mobile phase solutions of 10 mM ammonium acetate in 50:50 and 95:5 acetonitrile:water ratios. In the case of RPLC, a Zorbax SBaq column was used, and the mobile phase consisted of 0.06% acetic acid in water and methanol. Metabolic feature detection was performed using Progenesis QI software. Features from blanks and those lacking sufficient linearity upon dilution were excluded. Only features appearing in more than 33% of the samples were retained for subsequent analyses, and any missing values were imputed using the *k*-nearest neighbors approach. We employed locally estimated scatterplot smoothing (LOESS) normalization^83^ to correct the metabolite-specific signal drift over time. The metid package^84^ was used for metabolite annotation.

### Cytokine data

A panel of 62 human cytokines, chemokines and growth factors was analyzed in EDTA-anticoagulated plasma samples using Luminex-based multiplex assays with conjugated antibodies (Affymetrix). Raw fluorescence measurements were standardized to median fluorescence intensity values and subsequently subjected to variance-stabilizing transformation to account for batch-related variations. As previously reported^76^, data points characterized by background noise, termed CHEX, that deviate beyond five standard deviations from the mean (mean ± 5 × s.d.) were excluded from the analyses.

### Clinical laboratory test

The tests encompassed a comprehensive metabolic panel, a full blood count, glucose and HbA1C levels, insulin assays, high-sensitivity C-reactive protein (hsCRP), immunoglobulin M (IgM) and lipid, kidney and liver panels.

### Lipidomics

Lipid extraction and quantification procedures were executed in accordance with established protocols^78^. In summary, complex lipids were isolated from 40 μl of EDTA plasma using a solvent mixture comprising methyl tertiary-butyl ether, methanol and water, followed by a biphasic separation. Subsequent lipid analysis was conducted on the Lipidyzer platform, incorporating a differential mobility spectrometry device (SelexION Technology) and a QTRAP 5500 mass spectrometer (SCIEX).

### Microbiome

Immediately after arrival, samples were stored at −80 °C. Stool and nasal samples were processed and sequenced in-house at the Jackson Laboratory for Genomic Medicine, whereas oral and skin samples were outsourced to uBiome for additional processing. Skin and oral samples underwent 30 min of beads-beating lysis, followed by a silica-guanidinium thiocyanate-based nucleic acid isolation protocol. The V4 region of the 16S rRNA gene was amplified using specific primers, after which the DNA was barcoded and sequenced on an Illumina NextSeq 500 platform via a 2 × 150-bp paired-end protocol. Similarly, stool and nasal samples were processed for 16S rRNA V1–V3 region amplification using a different set of primers and sequenced on an Illumina MiSeq platform. For data processing, the raw sequencing data were demultiplexed using BCL2FASTQ software and subsequently filtered for quality. Reads with a Q-score lower than 30 were excluded. The DADA2 R package was used for further sequence data processing, which included filtering out reads with ambiguous bases and errors, removing chimeras and aligning sequences against a validated 16S rRNA gene database. Relative abundance calculations for amplicon sequence variants (ASVs) were performed, and samples with inadequate sequencing depth (<1,000 reads) were excluded. Local outlier factor (LOF) was calculated for each point on a depth-richness plot, and samples with abnormal LOF were removed. In summary, rigorous procedures were followed in both the collection and processing stages, leveraging automated systems and specialized software to ensure the quality and integrity of the microbiome data across multiple body sites.

### Statistics and reproducibility

For all data processing, statistical analysis and data visualization tasks, RStudio, along with R language (v.4.2.1), was employed. A comprehensive list of the packages used can be found in the Supplementary Note. The Benjamini–Hochberg method was employed to account for multiple comparisons. Spearman correlation coefficients were calculated using the R functions ‘cor’ and ‘cor.test’. Principal-component analysis (PCA) was conducted using the R function ‘princomp’. Before all the analyses, the confounders, such as BMI, sex, IRIS and ethnicity, were adjusted using the previously published method^19^. In brief, we used the intensity of each feature as the dependent variable (Y) and the confounding factors as the independent variables (X) to build a linear regression model. The residuals from this model were then used as the adjusted values for that specific feature.

All the omics data were acquired randomly. No statistical methods were used to predetermine the sample size, but our sample sizes are similar to those reported in previous publications^5,76–79^, and no data were excluded from the analyses. Additionally, the investigators were blinded to allocation during experiments and outcome assessment to the conditions of the experiments. Data distribution was assumed to be normal, but this was not formally tested.

The icons used in figures are from iconfont.cn, which can be used for non-commercial purposes under the MIT license (https://pub.dev/packages/iconfont/license).

### Cross-sectional dataset generation

The ‘cross-sectional’ dataset was created by briefly extracting information from the longitudinal dataset. The mean value was calculated to represent each molecule’s intensity for each participant. Similarly, the age of each participant was determined by calculating the mean value of ages across all sample collection time points.

### Linear changing molecule detection

We detected linear changing molecules during human aging using Spearman correlation and linear regression modeling. The confounders, such as BMI, sex, IRIS and ethnicity, were adjusted using the previously published method^19^. Our analysis revealed a high correlation between these two approaches in identifying such molecules. Based on these findings, we used the Spearman correlation approach to showcase the linear changing molecules during human aging. The permutation test was also used to get the permutated *P* values for each feature. In brief, each feature was subjected to sample label shuffling followed by a recalculation of the Spearman correlation. This process was reiterated 10,000 times, yielding 10,000 permuted Spearman correlations. The original Spearman correlation was then compared against these permuted values to obtain the permuted *P* values.

### Dysregulated molecules compared to baseline during human aging

To depict the dysregulated molecules during human aging compared to the baseline, we categorized the participants into different age stages based on their ages. The baseline stage was defined as individuals aged 25–40 years. For each age stage group, we employed the Wilcoxon test to identify dysregulated molecules in comparison to the baseline, considering a significance threshold of *P* < 0.05. Before the statistical analysis, all the confounders were corrected. Subsequently, we visualized the resulting dysregulated molecules at different age stages using a Sankey plot. The permutation test was also used to get the permutated *P* values for each feature. In brief, we shuffled the sample labels and recalculated the absolute mean difference between the two groups, against which the actual absolute mean difference was benchmarked to derive the permuted *P* values. To identify the molecules and microbes that exhibited significant changes at any given age stage, we adjusted the *P* values for each feature by multiplying them by 6. This adjustment adheres to the Bonferroni correction method, ensuring a rigorous evaluation of statistical significance.

### Evaluation of the age reflected by different types of omics data

To assess whether each type of omics data accurately reflects the ages of individuals in our dataset, we conducted a PCA. Subsequently, we computed the Spearman correlation coefficient between the ages of participants and the first principal component (PC1). The absolute value of this coefficient was used to evaluate the degree to which the omics data reflect the ages (Fig. 2a). PLS regression was also used to compare the strength of the age effect to the different omics data types. In brief, the ‘pls’ function from the R package mixOmics was used to construct the regression model between omics data and ages. Then, the ‘perf’ function was used to assess the performance of all the modules with sevenfold cross-validation. The *R*^2^ was extracted to assess the strength of the age effect on the different omics data types.

### LOESS data

To accommodate the varying time points of biological and omics data, we employed the LOESS approach. This approach allowed us to smooth and predict the multi-omics data at specific time points (that is, every half year)^14,85^. In brief, for each molecule, we fitted a LOESS regression model. During the fitting process, the LOESS argument ‘span’ was optimized through cross-validation. This ensured that the LOESS model provided an accurate and non-overfitting fit to the data (Supplementary Fig. 2a,b). Once we obtained the LOESS prediction model, we applied it to predict the intensity of each molecule at every half-year time point.

### Trajectory clustering analysis

To conduct trajectory clustering analysis, we employed the fuzzy c-means clustering approach available in the R package ‘Mfuzz.’ This approach was previously described in our publication^19^. The analysis proceeded in several steps. First, the omics data were auto-scaled to ensure comparable ranges. Next, we computed the minimum centroid distances for a range of cluster numbers, specifically from 2 to 22, in step 1. These minimum centroid distances served as a cluster validity index, helping us determine the optimal cluster number. Based on predefined rules, we selected the optimal cluster number. To refine the accuracy of this selection, we merged clusters with center expression data correlations greater than 0.8 into a single cluster. This step aimed to capture similar patterns within the data. The resulting optimal cluster number was then used for the fuzzy c-means clustering. Only molecules with memberships above 0.5 were retained within each cluster for further analysis. This threshold ensured that the molecules exhibited a strong association with their assigned cluster and contributed considerably to the cluster’s characteristics.

### Pathway enrichment analysis and functional module identification

#### Transcriptomics and proteomics pathway enrichment

Pathway enrichment analysis was conducted using the ‘clusterProfiler’ R package^86^. The GO, KEGG and Reactome databases were used. The *P* values were adjusted using the Benjamini–Hochberg method, with a significance threshold set at <0.05. To minimize redundant enriched pathways and GO terms, we employed a series of analyses. First, for enriched GO terms, we used the ‘Wang’ algorithm from the R package ‘simplifyEnrichment’ to calculate the similarity between GO terms. Only connections with a similarity score greater than 0.7 were retained to construct the GO term similarity network. Subsequently, community analysis was performed using the ‘igraph’ R package to partition the network into distinct modules. The GO term with the smallest enrichment adjusted *P* value was chosen as the representative within each module. The same approach was applied to the enriched KEGG and Reactome pathways, with one slight modification. In this case, the ‘jaccard’ algorithm was used to calculate the similarity between pathways, and a similarity cutoff of 0.5 was employed for the Jaccard index. After removing redundant enriched pathways, we combined all the remaining GO terms and pathways. Subsequently, we calculated the similarity between these merged entities using the Jaccard index. This similarity analysis aimed to capture the overlap and relationships between the different GO terms and pathways. Using the same approach as before, we performed community analysis to identify distinct biological functional modules based on the merged GO terms and pathways.

#### Identification of functional modules

First, we used the ‘Wang’ algorithm for the GO database and the ‘jaccard’ algorithm for the KEGG and Reactome databases to calculate the similarity between pathways. The enriched pathways served as nodes in a similarity network, with edges representing the similarity between two nodes. Next, we employed the R package ‘igraph’ to identify modules within the network based on edge betweenness. By gradually removing edges with the highest edge betweenness scores, we constructed a hierarchical map known as a dendrogram, representing a rooted tree of the graph. The leaf nodes correspond to individual pathways, and the root node represents the entire graph^87^. We then merged pathways within each module, selecting the pathway with the smallest adjusted *P* value to represent the module. After this step, we merged pathways from all three databases into modules. Subsequently, we repeated the process by calculating the similarity between modules from all three databases using the ‘jaccard’ algorithm. Once again, we employed the same approach described above to identify the functional modules.

#### Metabolomics pathway enrichment

To perform pathway enrichment analysis for metabolomics data, we used the human KEGG pathway database. This database was obtained from KEGG using the R package ‘massDatabase’^88^. For pathway enrichment analysis, we employed the hypergeometric distribution test from the ‘TidyMass’ project^89^. This statistical test allowed us to assess the enrichment of metabolites within each pathway. To account for multiple tests, *P* values were adjusted using the Benjamini–Hochberg method. We considered pathways with Benjamini–Hochberg-adjusted *P* values lower than 0.05 as significantly enriched.

### Modified DE-SWAN

The DE-SWAN algorithm^14^ was used. To begin, a unique age is selected as the center of a 20-year window. Molecule levels in individuals younger than and older than that age are compared using the Wilcoxon test to assess differential expression. *P* values are calculated for each molecule, indicating the significance of the observed differences. To ensure sufficient sample sizes for statistical analysis in each time window, the initial window ranges from ages 25 to 50. The left half of this window covers ages 25–40, whereas the right half spans ages 41–50. The window then moves in one-year steps; this is why Fig. 4 displays an age range of 40–65 years. To account for multiple comparisons, these *P* values are adjusted using Benjamini–Hochberg correction. To evaluate the robustness and relevance of the DE-SWAN results, the algorithm is tested with various parcel widths, including 15 years, 20 years, 25 years and 30 years. Additionally, different *q* value thresholds, such as <0.0001, <0.001, <0.01 and <0.05, are applied. By comparing the results obtained with these different parameters to results obtained by chance, we can assess the significance of the findings. To generate random results for comparison, the phenotypes of the individuals are randomly permuted, and the modified DE-SWAN algorithm is applied to the permuted dataset. This allows us to determine whether the observed results obtained with DE-SWAN are statistically significant and not merely a result of chance.

### Reporting summary

Further information on research design is available in the Nature Portfolio Reporting Summary linked to this article.

## Supplementary information

Supplementary Figs. 1–6

Reporting Summary

Supplementary data analysis results of the study.

## Data Availability

The raw data used in this study can be accessed without any restrictions on the National Institutes of Health Human Microbiome 2 project site (https://portal.hmpdacc.org). Both the raw and processed data are also available on the Stanford iPOP site (http://med.stanford.edu/ipop.html). Researchers and interested individuals can visit these websites to access the data. For further details and inquiries about the study, we recommend contacting the corresponding author, who can provide additional information and address any specific questions related to the research.

## Code availability

The statistical analysis and data processing in this study were performed using R v.4.2.1, along with various base packages and additional packages. Detailed information about the specific packages used can be found in the Supplementary Note, which accompanies the manuscript. Furthermore, all the custom scripts developed for this study have been made openly accessible and can be found on the GitHub repository at https://github.com/jaspershen-lab/ipop_aging. By visiting this repository, researchers and interested individuals can access and use the custom scripts for their own analyses or to replicate the study’s findings.
