# Supplementary material for: Nonlinear dynamics of multi-omics profiles during human aging
Source: Nat Aging. 2024 Aug 14;4(11):1619–34. doi: 10.1038/s43587-024-00692-2 (PMC11564093; doi:10.1038/s43587-024-00692-2)
Supplement: Supplementary file 1 — Supplementary Figs. 1–6 [file 43587_2024_692_MOESM1_ESM.pdf]

nature aging

Supplementary information https://doi.org/10.1038/s43587-024-00692-2

Nonlinear dynamics of multi-omics profiles during human aging

In the format provided by the authors and unedited

Table of Contents

Supplementary Figure 1. Linear changing molecules during human aging.

Supplementary Figure 2. LOESS smoothing for omics data and Fuzzy C-clustering.

Supplementary Figure 3. Waves of molecules and microbes during aging.

Supplementary Figure 4. DE-SWAN age effect for multiple *q*-values cutoffs, windows size, and after phenotypes permutations.

Supplementary Figure 5. The overlap of all types of omics data between crests 1 and 2.

Supplementary Note. R Packages used in the study

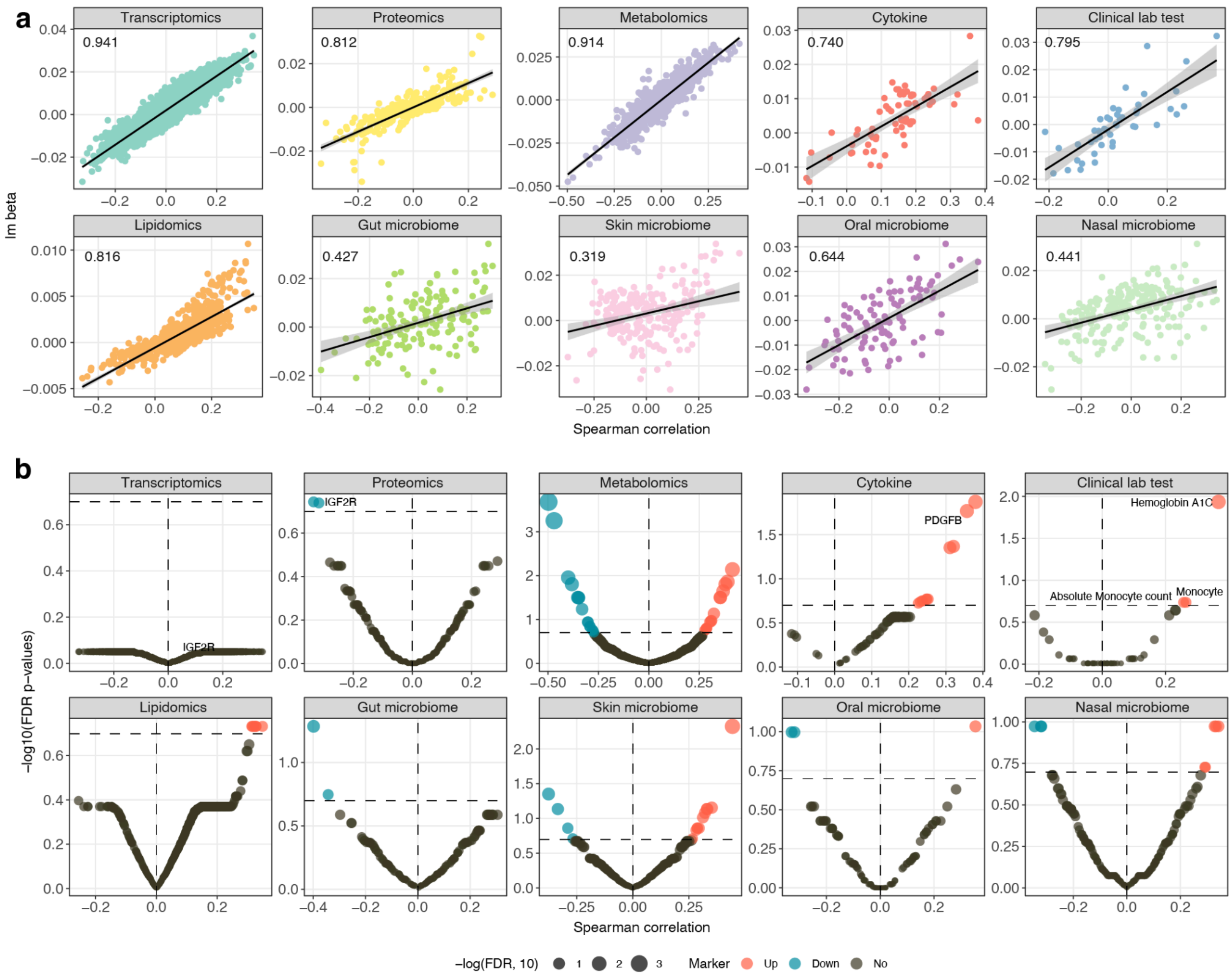

Supplementary Figure 1. Linear changing molecules during human aging. a, The Spearman correlations highly correlate with the beta coefficients from linear regression models for each type of omics data. The shaded area around the regression line represents the 95% confidence interval. **b,** Linear changing molecules for different types of omics data using the Spearman correlation approach (FDR-adjusted *p-values* < 0.05).

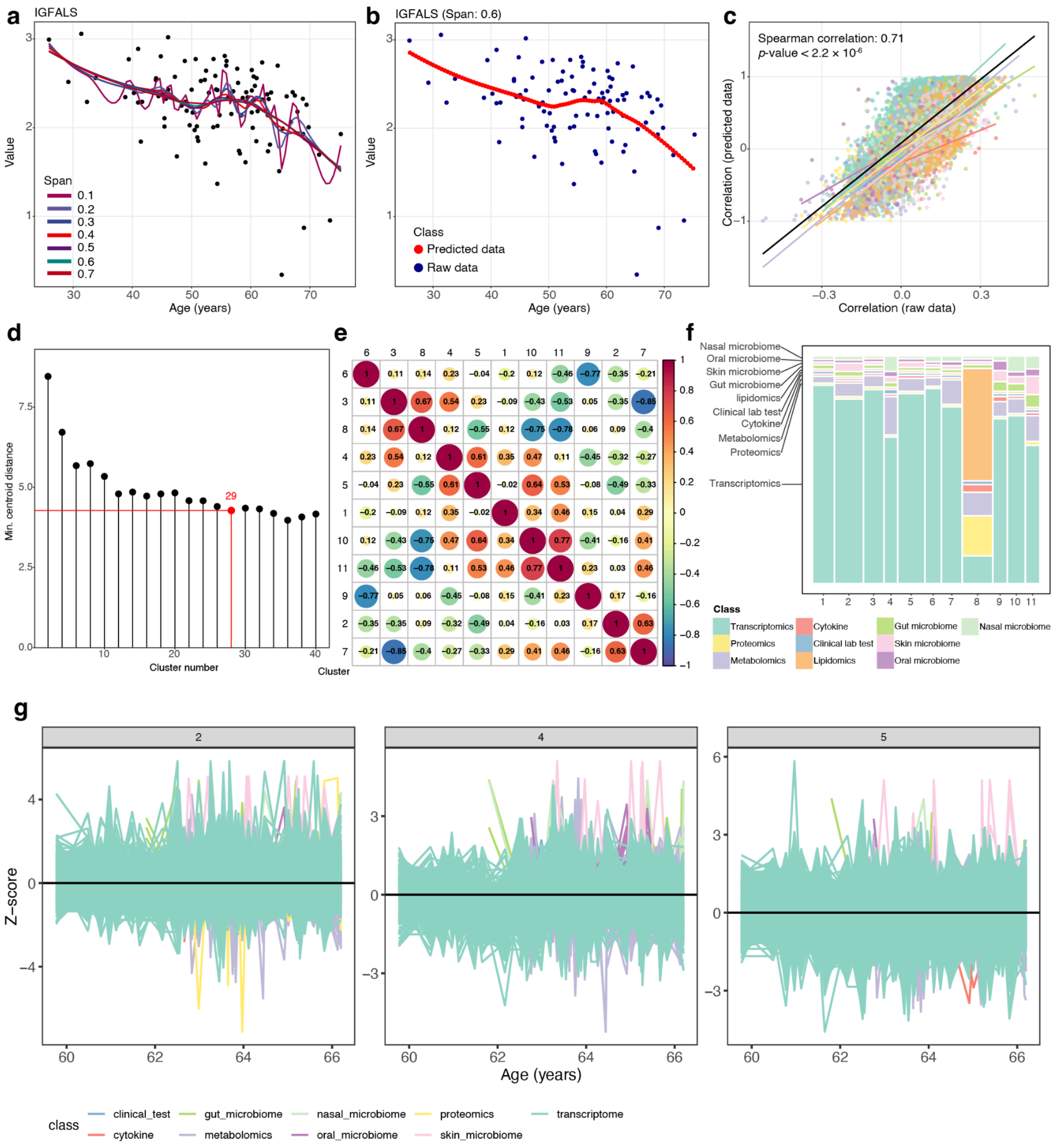

Supplementary Figure 2. LOESS smoothing for omics data and Fuzzy C-clustering. a, LOESS smoothing for protein IGFALS with parameter Span from 0.1 to 0.7 (step = 0.1). **b,** LOESS smoothing for protein IGFALS with best span 0.6. **c,** The predicted data and raw data have high correlations for all the omics data (Spearman correlation: 0.71, p-value < 2.2 × 10^{-6}). **d,** The optimization of the number of clusters. **e,** The correlation matrix of 11 clusters for all the omics data. **f,** The classes of all the molecules in all the clusters. **g,** The molecules in clusters 2, 4, and 5 at individual levels.

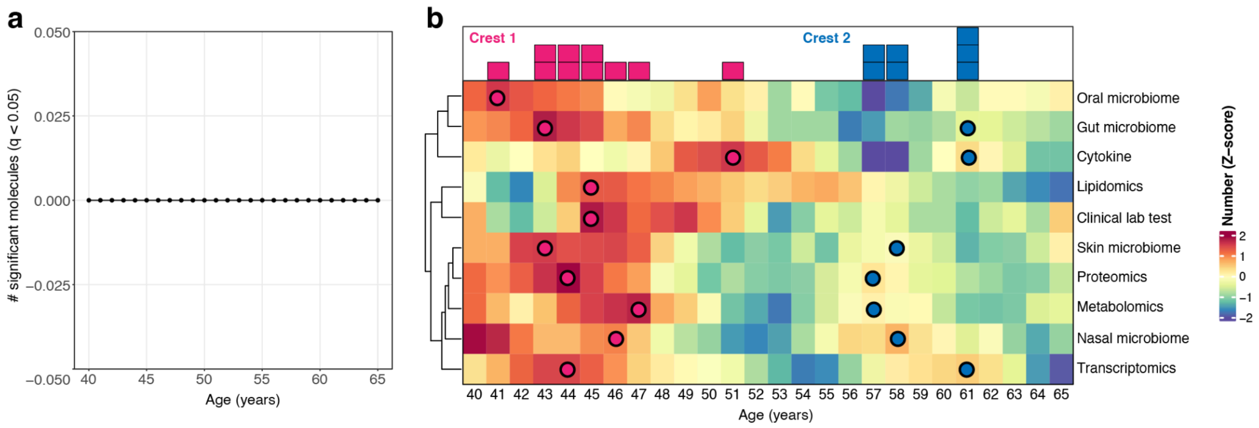

Supplementary Figure 3. Waves of molecules and microbes during aging. a, The waves disappeared when the phenotypes of the individuals were randomly permuted. **b**, The waves for different types of omics data across the human lifespan.

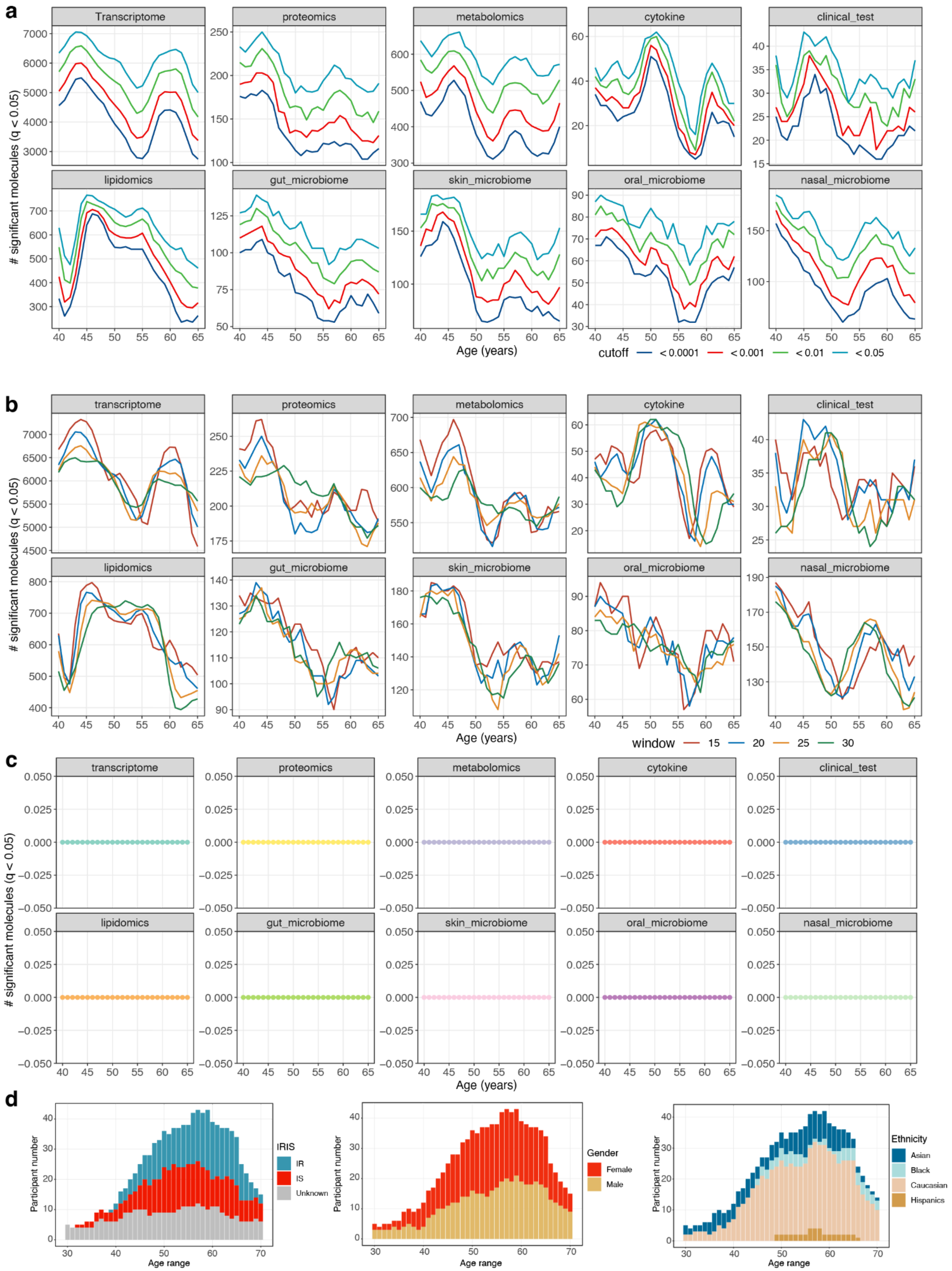
a
Transcriptome
proteomics
metabolomics
cytokine
clinical_test
lipidomics
gut_microbiome
skin_microbiome
oral_microbiome
nasal_microbiome
significant molecules (q < 0.05)
Age (years)
cutoff < 0.0001 < 0.001 < 0.01 < 0.05
b
window 15 20 25 30
c
d
Participant number
Age range
IRIS
IR
IS
Unknown
Gender
Female
Male
Ethnicity
Asian
Black
Caucasian
Hispanics

Supplementary Figure 4. DE-SWAN age effect for multiple *q*-values cutoffs, windows size, and after phenotypes permutations. a, Different *q*-value cutoffs are used for each type of omics data, respectively. **b,** Different windows are used for each type of omics data. **c,** The crests disappeared when the sample labels were randomly shifted for each type of omics data respectively. **d,** The distribution of insulin sensitivity, gender, and ethnicity across ages.

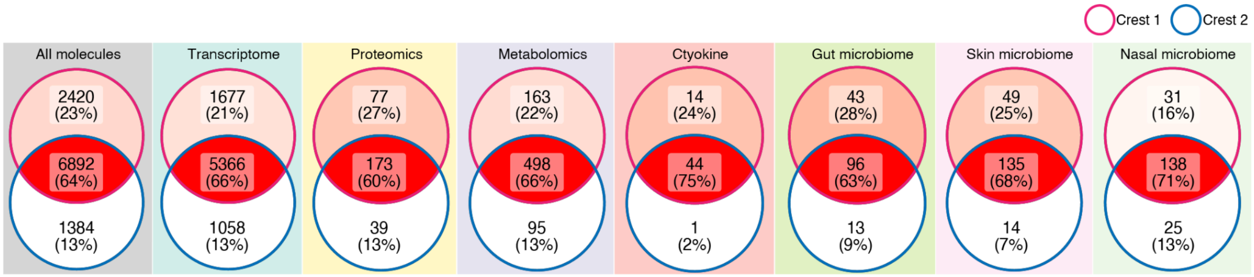

Supplementary Figure 5. The overlap of all types of omics data between crests 1 and 2.

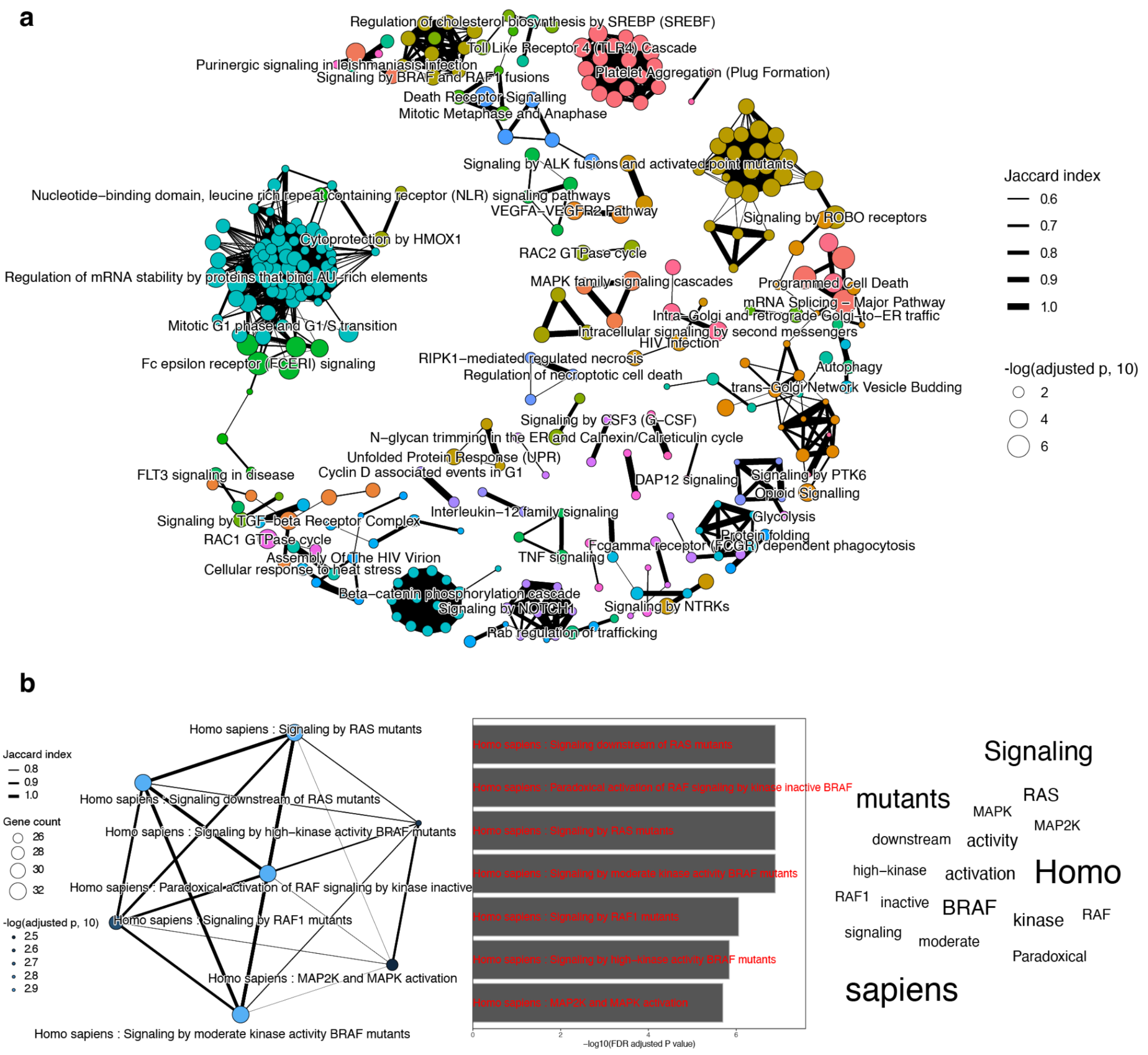

Supplementary Figure 6. The community analysis to reduce the redundancy of enriched GO terms and pathways. a, The similarity network and molecule detection. **b,** One example module detected from the similarity network.

Supplementary Note

R Packages used in the study

[1] stats graphics grDevices utils datasets methods base

loaded via a namespace (and not attached):

[1] tidyselect_1.2.0 viridisLite_0.4.2 dplyr_1.1.2

[4] farver_2.1.1 viridis_0.6.3 ggraph_2.1.0

[7] tweenr_2.0.2 XML_3.99-0.14 digest_0.6.31

[10] lifecycle_1.0.3 cluster_2.1.4 ProtGenerics_1.32.0

[13] magrittr_2.0.3 compiler_4.3.0 rlang_1.1.1

[16] tools_4.3.0 igraph_1.4.3 utf8_1.2.3

[19] graphlayouts_1.0.0 plyr_1.8.8 BiocParallel_1.34.2

[22] withr_2.5.0 purrr_1.0.1 BiocGenerics_0.46.0

[25] grid_4.3.0 polyclip_1.10-4 stats4_4.3.0

[28] preprocessCore_1.62.1 fansi_1.0.4 colorspace_2.1-0

[31] extrafontdb_1.0 ggplot2_3.4.2 scales_1.2.1

[34] iterators_1.0.14 MASS_7.3-58.4 cli_3.6.1

[37] mzR_2.34.0 crayon_1.5.2 generics_0.1.3

[40] remotes_2.4.2 Rdisop_1.60.0 rstudioapi_0.14

[43] readxl_1.4.2 ncdf4_1.21 pbapply_1.7-0

[46] affy_1.78.0 ggforce_0.4.1 stringr_1.5.0

[49] zlibbioc_1.46.0 parallel_4.3.0 ggplotify_0.1.0

[52] impute_1.74.1 cellranger_1.1.0 BiocManager_1.30.21

[55] vsn_3.68.0 yulab.utils_0.0.6 vctrs_0.6.2

[58] shadowtext_0.1.2 gridGraphics_0.5-1 IRanges_2.34.0

[61] S4Vectors_0.38.1 MALDIquant_1.22.1 ggrepel_0.9.3

[64] clue_0.3-64 foreach_1.5.2 limma_3.56.2

[67] tidyr_1.3.0 affyio_1.70.0 ggVennDiagram_1.2.2

[70] glue_1.6.2 MSnbase_2.26.0 codetools_0.2-19

[73] stringi_1.7.12 gtable_0.3.3 RVenn_1.1.0

[76] mzID_1.38.0 extrafont_0.19 munsell_0.5.0

[79] tibble_3.2.1 pillar_1.9.0 pcaMethods_1.92.0

[82] R6_2.5.1 doParallel_1.0.17 tidygraph_1.2.3

[85] lattice_0.21-8 Biobase_2.60.0 masstools_1.0.10

[88] openxlsx_4.2.5.2 ggsci_3.0.0 zip_2.3.0

[91] Rcpp_1.0.10 gridExtra_2.3 Rttf2pt1_1.3.12

[94] MsCoreUtils_1.12.0 pkgconfig_2.0.3
