# Supplementary material for: Nonlinear dynamics of multi-omics profiles during human aging
Source: Nat Aging. 2024 Aug 14;4(11):1619–34. doi: 10.1038/s43587-024-00692-2 (PMC11564093; doi:10.1038/s43587-024-00692-2)
Supplement: Supplementary file 2 — Reporting Summary [file 43587_2024_692_MOESM2_ESM.pdf]

Corresponding author(s): Michael P. Snyder

Last updated by author(s): Jun 12, 2024

Reporting Summary

Nature Portfolio wishes to improve the reproducibility of the work that we publish. This form provides structure for consistency and transparency in reporting. For further information on Nature Portfolio policies, see our Editorial Policies and the Editorial Policy Checklist.

Statistics

For all statistical analyses, confirm that the following items are present in the figure legend, table legend, main text, or Methods section.

n/a	Confirmed	
☐	☒	The exact sample size (*n*) for each experimental group/condition, given as a discrete number and unit of measurement
☐	☒	A statement on whether measurements were taken from distinct samples or whether the same sample was measured repeatedly
☐	☒	The statistical test(s) used AND whether they are one- or two-sided<br>*Only common tests should be described solely by name; describe more complex techniques in the Methods section.*
☐	☒	A description of all covariates tested
☐	☒	A description of any assumptions or corrections, such as tests of normality and adjustment for multiple comparisons
☐	☒	A full description of the statistical parameters including central tendency (e.g. means) or other basic estimates (e.g. regression coefficient) AND variation (e.g. standard deviation) or associated estimates of uncertainty (e.g. confidence intervals)
☐	☒	For null hypothesis testing, the test statistic (e.g. F, t, r) with confidence intervals, effect sizes, degrees of freedom and P value noted<br>*Give P values as exact values whenever suitable.*
☒	☐	For Bayesian analysis, information on the choice of priors and Markov chain Monte Carlo settings
☒	☐	For hierarchical and complex designs, identification of the appropriate level for tests and full reporting of outcomes
☒	☐	Estimates of effect sizes (e.g. Cohen's d, Pearson's r), indicating how they were calculated

Our web collection on statistics for biologists contains articles on many of the points above.

Software and code

Policy information about availability of computer code

Data collection	ProteoWizard: Version. 3.0.19314-fb982f15b
Data analysis	R: 4.1.2; Rstudio: 2021.09.2. R package: colorspace_2.0-2 rjson_0.2.21 ellipsis_0.3.2 leaflet_2.1.0 rprojroot_2.0.2 circlize_0.4.14 GlobalOptions_0.1.2 clue_0.3-60 rstudioapi_0.13 mzR_2.28.0 affyio_1.64.0 fansi_1.0.2 xml2_1.3.3 codetools_0.2-18 ncdf4_1.19 doParallel_1.0.17 impute_1.68.0 knitr_1.37 jsonlite_1.7.3 cluster_2.1.2 vsn_3.62.0 png_0.1-7 readr_2.1.2 compiler_4.1.2 httr_1.4.2 assertthat_0.2.1 fastmap_1.1.0 lazyeval_0.2.2 limma_3.50.0 cli_3.2.0 htmltools_0.5.2 tools_4.1.2 gtable_0.3.0 glue_1.6.1 affy_1.72.0 dplyr_1.0.8 Biobase_2.54.0 cellranger_1.1.0 jquerylib_0.1.4 iterators_1.0.14 crosstalk_1.2.0 stringr_1.4.0 openxlsx_4.2.5 MSnbase_2.20.4 pcaMethods_1.86.0 hms_1.1.1 ProtGenerics_1.26.0 parallel_4.1.2 RColorBrewer_1.1-2 ComplexHeatmap_2.10.0 yaml_2.3.4 pbapply_1.5-0 yulab.utils_0.0.4 sass_0.4.0 stringi_1.7.6 highr_0.9 S4Vectors_0.32.3 foreach_1.5.2 BiocGenerics_0.40.0 zip_2.2.0 BiocParallel_1.28.3 shape_1.4.6 systemfonts_1.0.3 rlang_1.0.1 pkgconfig_2.0.3 matrixStats_0.61.0 mzID_1.32.0 evaluate_0.15 lattice_0.20-45 purrr_0.3.4 htmlwidgets_1.5.4 tidyselect_1.1.1 here_1.0.1 ggsci_2.9 plyr_1.8.6 bookdown_0.24 R6_2.5.1 IRanges_2.28.0 generics_0.1.2 DBI_1.1.2 pillar_1.7.0 withr_2.4.3 MsCoreUtils_1.6.0 tibble_3.1.6 crayon_1.5.0 utf8_1.2.2 plotly_4.10.0 tzdb_0.2.0 readxl_1.3.1 data.table_1.14.2 webshot_0.5.2 digest_0.6.29 tidyr_1.2.0 gridGraphics_0.5-1 ggplotify_0.1.0 bslib_0.3.1

For manuscripts utilizing custom algorithms or software that are central to the research but not yet described in published literature, software must be made available to editors and reviewers. We strongly encourage code deposition in a community repository (e.g. GitHub). See the Nature Portfolio guidelines for submitting code & software for further information.

Data

Policy information about availability of data

All manuscripts must include a data availability statement. This statement should provide the following information, where applicable:

- Accession codes, unique identifiers, or web links for publicly available datasets
- A description of any restrictions on data availability
- For clinical datasets or third party data, please ensure that the statement adheres to our policy

The raw data used in this study can be accessed without any restrictions on the NIH Human Microbiome 2 project site (https://portal.hmpdacc.org). Both the raw and processed data are also available on the Stanford iPOP site (http://med.stanford.edu/ipop.html). Researchers and interested individuals can visit these websites to access the data. For further details and inquiries about the study, we recommend contacting the corresponding author, who can address any specific questions related to the research. GO (https://geneontology.org/), KEGG (https://www.genome.jp/kegg/) and Reactome (https://reactome.org/) databases were used in the study.

Research involving human participants, their data, or biological material

Policy information about studies with human participants or human data. See also policy information about sex, gender (identity/presentation), and sexual orientation and race, ethnicity and racism.

Reporting on sex and gender	We have reported the sex information on the enrolled patients, which is determined by self-reporting
Reporting on race, ethnicity, or other socially relevant groupings	We have reported the race and ethnicity information on the enrolled patients, which is determined by self-reporting. Before all the analysis, the confounders were adjusted using the previously published method. In brief, we used the intensity of each feature as the dependent variable (Y) and the confounding factors as the independent variables (X) to build a linear regression model. The residuals from this model were then used as the adjusted values for that specific feature.
Population characteristics	All the participants had diverse backgrounds and ages ranging from 25 - 75 years (median: 55.7 years). The Body Mass Index (BMI) of the participants ranged from 19.1 to 40.83 (median: 28.24). 51.9% of the participants were female.
Recruitment	We tracked 108 participants for up to 6.8 years with longitudinal biological sample collection and multi-omics profiling. The participants were sampled every 3-6 months while healthy. All the participants have no compensation.
Ethics oversight	Stanford University Institutional Review Board (IRB 23602)

Note that full information on the approval of the study protocol must also be provided in the manuscript.

Field-specific reporting

Please select the one below that is the best fit for your research. If you are not sure, read the appropriate sections before making your selection.

☒ Life sciences ☐ Behavioural & social sciences ☐ Ecological, evolutionary & environmental sciences

For a reference copy of the document with all sections, see nature.com/documents/nr-reporting-summary-flat.pdf

Life sciences study design

All studies must disclose on these points even when the disclosure is negative.

Sample size	No statistical methods were used to calculate the sample size. 108 participants were enrolled. For each participant, the omics data were aggregated and averaged across all healthy samples to represent the individual's mean value. Compared to cross-sectional cohorts, which have only a one-time point sample from each participant, our longitudinal dataset, which includes multiple time-point samples from each participant, is more robust for detecting complex aging-related changes in molecules and functions. This is because analysis of multi-time-point samples can detect participants' baseline and robustly evaluate individuals' longitudinal molecular changes.
Data exclusions	As frequently used in microbiome studies, for microbiome analyses, to capture major changes across the whole cohort, we focused on prevalent taxa and genes out of all mapped ones, excluding those that were present in less than half of the cohort.
Replication	This was an observational study which did not involve experiments.
Randomization	Samples were randomly arranged by computing program into various batches of omics assay.
Blinding	Each sample was labeled with an numeric ID whose annotation was kept blinded during data collection and analyses.

Reporting for specific materials, systems and methods

We require information from authors about some types of materials, experimental systems and methods used in many studies. Here, indicate whether each material, system or method listed is relevant to your study. If you are not sure if a list item applies to your research, read the appropriate section before selecting a response.

Materials & experimental systems

n/a	Involved in the study
☒	☐ Antibodies
☒	☐ Eukaryotic cell lines
☒	☐ Palaeontology and archaeology
☒	☐ Animals and other organisms
☒	☐ Clinical data
☒	☐ Dual use research of concern
☒	☐ Plants

Methods

n/a	Involved in the study
☒	☐ ChIP-seq
☒	☐ Flow cytometry
☒	☐ MRI-based neuroimaging
